# Polyphasic taxonomy of entomopathogenic fungi infecting scale insects in *Clavicipitaceae (Hypocreales)* from China

**DOI:** 10.3897/imafungus.17.195753

**Published:** 2026-06-23

**Authors:** Feng Liu, Feihu Wang, Xiulan Xu, Shasha Xiang, Xinyue Li, Xuejing Jiang, Hua Yang, Long Liu, Yinggao Liu, Shujiang Li, Chunlin Yang

**Affiliations:** 1 College of Forestry, Sichuan Agricultural University, Chengdu 611130, Sichuan, China Bamboo Resource Conservation and Utilization Key Laboratory of Sichuan Province, College of Forestry, Sichuan Agricultural University Chengdu China; 2 Forest Ecology and Conservation in the Upper Reaches of the Yangtze River Key Laboratory of Sichuan Province, Chengdu 611130, Sichuan, China College of Forestry, Sichuan Agricultural University Chengdu China; 3 Sichuan Mt. Emei Forest Ecosystem National Observation and Research Station, Chengdu 611130, Sichuan, China Forest Ecology and Conservation in the Upper Reaches of the Yangtze River Key Laboratory of Sichuan Province Chengdu China; 4 Bamboo Resource Conservation and Utilization Key Laboratory of Sichuan Province, College of Forestry, Sichuan Agricultural University, Huiming Road 211, Wenjiang District, Chengdu 611130, Sichuan, China Sichuan Mt. Emei Forest Ecosystem National Observation and Research Station Chengdu China; 5 Forestry Research Institute, Chengdu Academy of Agricultural and Forestry Sciences, Chengdu 611130, Sichuan, China Forestry Research Institute, Chengdu Academy of Agricultural and Forestry Sciences Chengdu China

**Keywords:** *
Clavicipitaceae
*, fungal entomopathogens, new taxa, phylogeny, taxonomy

## Abstract

Entomopathogenic fungi in *Clavicipitaceae (Hypocreales)* serve as critical regulators of scale insect populations in agricultural and forest ecosystems, playing irreplaceable roles in natural pest control and exhibiting considerable potential for biological control applications. Despite their ecological importance, the species diversity and phylogenetic relationships of these fungi remain significantly understudied in Southwestern China, especially in Sichuan Province. In this study, we surveyed scale-insect pathogenic fungi across various forest ecosystems in Sichuan. Based on morphological observations and multi-locus phylogenetic analyses (LSU, *tef*1-α, *rpb*1), 24 specimens were classified into 9 species in 4 genera: *Conoideocrella*, *Hypocrella*, *Helicocollum*, *Regiocrella*, including 5 new species and 4 new provincial distribution records. Furthermore, this study proposes the synonym of *C. tiankengensis* under *C. luteorostrata*. Detailed comparisons and discussions of morphologically similar species and phylogenetically related taxa are provided. Comprehensive descriptions of phenotypic features, including fungal colony morphology, micromorphology, physiology, and ecology, are supplemented with illustrations and notes for all taxa. This study significantly enriches the species diversity and morphological knowledge of entomopathogenic fungi in *Clavicipitaceae* associated with scale insects in Southwest China, and provides new insights into their phylogeny, host associations, morphological evolution, and biological control of pest insects.

## Introduction

Entomopathogenic fungi (EPF) are microorganisms that induce lethal infections in arthropods (Litwin et al. 2020; Kankanamalage et al. 2025). As an ecologically diverse assemblage with broad representation across multiple fungal phyla, EPF exhibit remarkable evolutionary and functional diversification (Araújo and Hughes 2016). Phylogenetic studies have consistently demonstrated that entomopathogenic fungi are distributed across 11 of the 19 fungal phyla (Ownley et al. 2010; Vega et al. 2012; Araújo and Hughes 2016; Litwin et al. 2020; Wei et al. 2023; Gielen et al. 2024). To date, more than 1,800 EPF species belonging to more than 100 distinct genera have been formally described (Humber 2008; St. Leger and Wang 2010; Behie and Bidochka 2014; Ashraf et al. 2020; Gielen et al. 2024; Chen et al. 2025a, 2025b). Collectively, these fungi parasitize insects from 20 orders, with pathogenicity observed across nearly all host developmental stages (Wei et al. 2023).

EPF frequently trigger insect disease epidemics, and their strong parasitic capabilities play a crucial ecological role in natural ecosystems (Mains 1948; Vega et al. 2012). As a result, these fungi are being developed as biocontrol agents for sustainable agriculture and as safer alternatives to chemical insecticides (Ferron 1978; Araújo and Hughes 2016; Wei et al. 2023). Around 170 strains from 12 species of entomopathogenic fungi have been developed and commercialized (Ríos-Moreno et al. 2016; Zhang et al. 2020a, 2020b). Among these, *Beauveria bassiana* (Bals.-Criv.) Vuill. stands out as a broad-spectrum fungus with potent insecticidal properties (Que et al. 2019). Certain entomopathogenic fungi, such as *Beauveria*, *Akanthomyces*, *Lecanicillium*, and *Metarhizium*, function as endophytes, promoting plant growth and inducing systemic pathogen resistance (Ownley et al. 2010; Gurulingappa et al. 2011; Ríos-Moreno et al. 2016; Nicoletti and Becchimanzi 2020; Nishi et al. 2020, 2022). Species such as *Pochonia chlamydosporia* (Goddard) Zare & W. Gams and *Metarhizium anisopliae* (Metschn.) Sorokīn colonize the plant rhizosphere, enhancing plant growth by modulating rhizosphere microbial communities, nutrient uptake, hormone release, and stress signaling (Kerry 2000; Kabaluk and Ericsson 2007). Entomopathogenic fungi can produce a variety of secondary metabolites with insecticidal, antimicrobial, immunomodulatory, and cytotoxic properties, making them valuable resources for applications in human, veterinary, and agricultural fields (Hyde et al. 2019; Zhang et al. 2020a, 2020b).

*Hypocreales*, established by Engler and Prantl in 1897 with *Hypocreaceae* as the type family, is renowned for its diverse ecological roles and extensive distribution (Rehner and Samuels 1995; Rossman 1996). To date, it is the largest order in *Sordariomycetes* of *Ascomycota*, comprising 31 families (Wijayawardene et al. 2022; Hou et al. 2023; Perera et al. 2023; Xiao et al. 2023; Yang et al. 2023; Hyde et al. 2024b; Luangsa-ard et al. 2026; Wei et al. 2026; Zhao et al. 2026). Hypocrealean entomopathogenic fungi primarily parasitize arthropods, including insects and spiders, and form hemibiotrophic relationships with their hosts (Kepler et al. 2012; Araújo and Hughes 2016; Bihal et al. 2023; Wei et al. 2023). Most known entomopathogenic fungi originate from *Clavicipitaceae*, *Cordycipitaceae*, and *Ophiocordycipitaceae* (Araújo and Hughes 2016; Kankanamalage et al. 2025). As a new family encompassing both insect and fungal pathogens, *Polycephalomycetaceae* was recently established by Xiao et al. (2023), thereby expanding the taxonomic diversity of entomopathogenic fungi. Although entomopathogenic fungi are predominantly isolated from arthropod carcasses, phylogenetic analyses reveal their presence in a variety of other environments, including microinvertebrates, fungi, plants, soil, and other environmental resources (Kankanamalage et al. 2025). Notably, their non-infective stages primarily persist in the soil, making it an important reservoir for these fungi (Wei et al. 2023). This ecological diversity underscores the adaptability and ecological significance of entomopathogenic fungi in various ecosystems.

*Clavicipitaceae* is a highly diverse and widely distributed family in terrestrial ecosystems, encompassing diverse ecological roles as pathogens, endophytes, saprobes, symbionts, and parasites across a broad range of hosts, including plants, insects, fungi, and other invertebrates (Spatafora et al. 2007; Sung et al. 2007; Kepler et al. 2012; Schardl et al. 2013; Wei et al. 2023). The family currently comprises approximately 56 genera and over 750 species (Hyde et al. 2024b). Based on molecular phylogenetic evidence integrated with morphological characters such as stromatal texture, pigmentation, and form, Sung et al. (2007) clarified the delimitation of *Clavicipitaceae* from *Cordycipitaceae* and *Ophiocordycipitaceae*. Numerous species in *Clavicipitaceae* are entomopathogenic fungi specializing in scale insects and whiteflies, which are predominantly distributed in genera such as *Dussiella*, *Conoideocrella*, *Helicocollum*, Hypocrella (Aschersonia), *Moelleriella*, *Orbiocrella*, *Regiocrella*, and *Samuelsia* (Sullivan et al. 2000; Chaverri et al. 2005a, 2005b; Chaverri et al. 2008; Rossman et al. 2016; Luangsa-ard et al. 2017; Chen et al. 2020; Yuan et al. 2020; Crous et al. 2024; Wang et al. 2024b; Chen et al. 2025a; Crous et al. 2025; Lin et al. 2025; Wang et al. 2025b). Additionally, recently established genera including *Paramoelleriella* and *Polymicrospora*, have been confirmed to be closely associated with scale insect hosts (Wang et al. 2025b). Although globally distributed, these fungi reach their highest diversity in tropical and subtropical regions, particularly in Southern and Southwestern China, where numerous new taxa have been discovered in recent years. However, the diversity of scale-insect-associated *Clavicipitaceae* remains poorly documented in Sichuan Province, and their host associations, morphological evolution, and phylogenetic relationships remain incompletely elucidated.

Extensive research by mycologists in Southwest China has led to the discovery of numerous new taxa of hypocrealean entomopathogenic fungi, significantly enhancing our understanding of their diversity (Mongkolsamrit et al. 2020; Wang et al. 2020; Xu et al. 2021; Liu et al. 2023; Tang et al. 2023; Wei et al. 2023; Xiao et al. 2023; Chuang et al. 2024; Chen et al. 2025a, 2025b; Yang et al. 2025). Additionally, numerous studies have investigated the taxonomy and biology of entomopathogenic fungi in biodiversity hotspots, particularly in regions such as Thailand and Chinese provinces (Yunnan, Guizhou and Taiwan). These efforts have led to a deeper understanding of fungal species diversity and their ecological associations with hosts and the environment (Luangsa-ard et al. 2018; Mongkolsamrit et al. 2020; Thanakitpipattana et al. 2020; Wang et al. 2020; Chuang et al. 2024). For instance, Wei et al. (2023) investigated the diversity of entomopathogenic fungi in Southwest China and Thailand, estimated the divergence times of key *Hypocreales* lineages, and provided insights into the distribution and evolutionary relationships among fungi, arthropods, microinvertebrates, and plant-associated fungi within clavicipitoids. Chen et al. (2025b) focused on the cryptic diversity of cordyceps-like fungi in the karst regions of Guizhou Province, China, identified abundant new taxa based on multi-gene phylogenetic and morphological evidence, uncovered high cryptic diversity within this typical karst ecosystem, and advanced our knowledge of the systematics and distribution of cordyceps-like fungi in Southwest China.

Sichuan Province, located in Southwest China, is characterized by unique geographical and ecological features, which support rich floral, faunal, and microbial diversity (Xu et al. 2017). For instance, 398 new fungal species have been formally described from this region between 2020 and 2024 alone, most of which were isolated from soil and plant substrates (Wang et al. 2021; Wang and Cai 2022, 2023; Wang et al. 2024a, 2025a). At present, unlike other groups of fungi such as phytopathogenic, endophytic, and mycorrhizal fungi, the knowledge of entomopathogenic fungal resources in Sichuan remains limited, with few systematic investigations and sustained research (De Wint et al. 2024; Hyde et al. 2024a). Among them, entomopathogenic fungi that parasitize scale insects have received relatively less attention.

During an investigation into the diversity of entomopathogenic fungi, 24 fresh specimens were collected from Sichuan Province, China. The study aims to: 1) employ a polyphasic approach combining morphological and molecular data to identify new specimens and isolates; 2) describe and illustrate novel species through phylogenetic and morphological analyses; 3) improve our understanding of the diversity, distribution, and host associations of entomopathogenic species that parasitize scale insects in Sichuan.

## Materials and methods

### Fungal materials and isolation

The specimens for this study were systematically collected from different regions of Sichuan Province during 2022–2025, spanning March to December. Entomopathogenic fungi were gathered from diverse habitats, including the forest floor, leaf litter, decayed wood, tree trunks, and both the undersides and upper surfaces of leaves. Each specimen was meticulously wrapped in aluminum foil to preserve the host, stored in sterile centrifuge tubes, and labeled with detailed information, including location, altitude, date, collector, and forest type. In addition, field photographs of fresh specimens were systematically captured using a Canon EOS760D camera to document habitat, stromatal color, and host characteristics whenever feasible. Following collection, the fungal samples were immediately stored at 4 °C in the laboratory for subsequent isolation. Axenic cultures were successfully established from germinating ascospores, conidia, or fungal stromal tissues in accordance with established protocols (Luangsa-ard et al. 2018; Mongkolsamrit et al. 2018). Fungal specimens were dried overnight at 45 °C using an electric thermostatic drier and deposited in the Herbarium of Sichuan Agricultural University, Chengdu, China (SICAU). The cultures were deposited at the Culture Collection in Sichuan Agricultural University (SICAUCC). Species’ names were registered in Index Fungorum (www.indexfungorum.org).

### Morphological observations

Before observing the macroscopic morphology of the entomopathogenic fungi samples, surface debris such as soil and plant material was carefully removed. The macro-morphological traits of the fungi, such as host association, stromatal color and shape, and perithecial orientation, were systematically documented. All observations were captured using a dissecting microscope (Nikon SMZ800N) to ensure precise documentation of the morphological features. Host species were identified with assistance from entomologists. The micro-morphological features, such as the shape and size of perithecia, asci, ascus caps, ascospores, part-spores, phialides, and conidia, were examined. Specimens were prepared in lactophenol cotton blue stain and examined with an Olympus BX53 compound microscope.

To examine and compare the morphological characteristics of conidia, phialides, and colony coloration, pure cultures were separately transferred onto three different media: potato dextrose agar (PDA, Difco: potato 200 g/L, dextrose 20 g/L, agar 15 g/L), quarter-strength Sabouraud dextrose agar with yeast extract (SDAY/4, Difco: dextrose 10 g/L, peptone 2.5 g/L, yeast extract 2.5 g/L, agar 15 g/L) (Bischoff et al. 2009), and oatmeal agar (OA, Difco: oatmeal 60 g/L, agar 15 g/L) (Mongkolsamrit et al. 2020). Cultures were incubated at 25 °C with alternating light and dark cycles (14 hours light, 10 hours dark) for 14 to 30 days. A minimum of 20 measurements were recorded for each key morphological structure, including conidia and phialides, using Image Framework (IFW 0.9.0.7).

### DNA extraction, PCR amplification, and sequencing

Genomic DNA was extracted from fresh specimens or fungal cultures using the New Plant Genomic DNA Kit (Beijing Aidlab Biotechnologies Co., Ltd, Beijing, China), and the extracted DNA was stored at −20 °C. Three nuclear DNA regions were amplified using PCR with primers: LR0R/LR5 for large subunit rRNA gene region (LSU) (Vilgalys and Hester 1990), 983F/2218R for translation elongation factor 1-alpha (*tef*1-α) (Rehner and Buckley 2005), and CRPB1A/RPB1Cr for the RNA polymerase II largest subunit (*rpb*1) (Castlebury et al. 2004), respectively. PCR amplifications were conducted in 25 µl reaction mixture comprising 22 µl Master Mix (Beijing LABLEAD Biotech Co., Ltd., Beijing, China), 1 µl DNA template, and 1 µl each of forward and reverse primers (10 µM). The PCR thermal cycling conditions for all three nuclear DNA regions adhered to the protocols outlined by Sung et al. (2007) and Xiao et al. (2023). The PCR products were sequenced by Hangzhou Youkang Biotech Co., Ltd., Chengdu, China, and the resulting sequences were submitted to GenBank with assigned accession numbers.

### Phylogenetic analyses

The newly generated DNA sequences were assembled and manually edited to resolve ambiguous bases using BioEdit version 7.2.5 (Hall 2011). All the sequences were aligned using BLASTn on the NCBI website to determine the approximate taxonomic unit of each species. Based on the latest research, a sequence set was selected for tree construction (Table 1), with reference sequences obtained from the GenBank database (Chaverri et al. 2005a, 2005b; Mongkolsamrit et al. 2016; Luangsa-ard et al. 2017; Lovett et al. 2022; Crous et al. 2024; Wang et al. 2024b; Crous et al. 2025; Wang et al. 2025b). Sequence alignments were performed for individual datasets using MAFFT v.7.526 and manually adjusted as needed (Katoh et al. 2019). SequenceMatrix v.1.7.8 (Vaidya et al. 2011) was used to concatenate datasets. Phylogenetic relationships were assessed through maximum likelihood (ML) and Bayesian inference (BI) analyses via the CIPRES Science Gateway (Miller et al. 2010). Taxonomic assignments, whether as new species or new provincial records, were based on the criteria outlined by Jeewon and Hyde (2016) and Chethana et al. (2021). The resulting phylogenetic tree was visualized using FigTree version 1.4.3, and further refined using Adobe Illustrator 2023 (Adobe Systems, San Jose, CA, USA).

**Table 1.** Voucher information and GenBank accession numbers of the taxa used in the *Clavicipitaceae*.

**Species**	**Voucher number**	**LSU**	***tef*1-α**	***rpb*1**	**References**
*Conoideocrella fenshuilingensis*	YHH CFFSL2310002 T	PP178583	PP776168	PP776158	Wang et al. (2024b)
*Conoideocrella fenshuilingensis*	YHH CFFSL2310003	PP178584	PP776169	PP776159	Wang et al. (2024b)
***Conoideocrella fenshuilingensis***	**SICAUCC 25-0180**	PZ205065	PZ221452	PZ221476	**This study**
*Conoideocrella gongyashanensis*	CGMCC 3.28305 T	PQ278801	PQ301442	PQ316534	Lin et al. (2025)
*Conoideocrella gongyashanensis*	CGMCC 3.28306	PQ278802	PQ301443	PQ316535	Lin et al. (2025)
***Conoideocrella gongyashanensis***	**SICAUCC 25-0185**	PZ205072	PZ221453	PZ221477	**This study**
***Conoideocrella gongyashanensis***	**SICAUCC 25-0186**	PZ205074	PZ221454	PZ221478	**This study**
***Conoideocrella gongyashanensis***	**SICAUCC 25-0187**	PZ205064	PZ221455	PZ221479	**This study**
***Conoideocrella jiufengensis***	**SICAUCC 24-0208 T**	**PV094302**	**PV268633**	**PV255821**	**This study**
***Conoideocrella jiufengensis***	**SICAUCC 24-0209**	**PV094303**	**PV268634**	**PV255822**	**This study**
***Conoideocrella jiufengensis***	**SICAUCC 24-0210**	**PV094304**	**PV268635**	**PV255823**	**This study**
***Conoideocrella jiufengensis***	**SICAUCC 25-0183**	PZ205073	PZ221456	PZ221480	**This study**
***Conoideocrella jiufengensis***	**SICAUCC 25-0184**	PZ205061	PZ221457	PZ221481	**This study**
*Conoideocrella krungchingensis*	BCC 36100 T	KJ435080	KJ435097	–	Mongkolsamrit et al. (2016)
*Conoideocrella krungchingensis*	BCC 36101	KJ435081	KJ435098	–	Mongkolsamrit et al. (2016)
*Conoideocrella luteorostrata*	NHJ 11343	EF468850	EF468801	EF468906	Kepler et al. (2012)
*Conoideocrella luteorostrata*	NHJ 12516	EF468849	EF468800	EF468905	Kepler et al. (2012)
***Conoideocrella luteorostrata***	**SICAUCC 25-0170**	PZ205078	PZ221458	PZ221482	**This study**
***Conoideocrella luteorostrata***	**SICAUCC 25-0171**	PZ205067	PZ221459	PZ221483	**This study**
***Conoideocrella luteorostrata***	**SICAUCC 25-0172**	PZ205070	PZ221460	PZ221484	**This study**
***Conoideocrella luteorostrata***	**SICAUCC 25-0173**	PZ205069	PZ221461	PZ221485	**This study**
***Conoideocrella luteorostrata***	**SICAUCC 25-0174**	PZ205079	PZ221462	PZ221486	**This study**
***Conoideocrella luteorostrata***	**SICAUCC 25-0175**	PZ205058	PZ221463	PZ221487	**This study**
***Conoideocrella luteorostrata***	**SICAUCC 25-0176**	PZ205071	PZ221464	PZ221488	**This study**
*Conoideocrella tenuis*	NHJ 6293	EU369044	EU369029	EU369068	Johnson et al. (2009)
*Conoideocrella tenuis*	NHJ 6791	EU369046	EU369028	EU369069	Johnson et al. (2009)
*Conoideocrella tenuis*	NHJ 345.01	EU369045	EU369030	–	Johnson et al. (2009)
***Conoideocrella tenuis***	**SICAUCC 25-0177**	PZ205066	PZ221465	PZ221489	**This study**
***Conoideocrella tenuis***	**SICAUCC 25-0178**	PZ205077	PZ221466	PZ221490	**This study**
***Conoideocrella tenuis***	**SICAUCC 25-0179**	PZ205068	PZ221467	PZ221491	**This study**
*Conoideocrella tiankengensis*	KY04071 T	PV688364	PV705692	–	Chen et al. (2025a)
*Conoideocrella tiankengensis*	KY04072	PV688365	PV705693	–	Chen et al. (2025a)
***Conoideocrella violaceomarginata***	**SICAUCC 25-0181 T**	PZ205059	PZ221468	PZ221492	**This study**
***Conoideocrella violaceomarginata***	**SICAUCC 25-0182**	PZ205060	PZ221469	PZ221493	**This study**
***Helicocollum bambusae***	**SICAUCC 25-0190 T**	PZ205062	PZ221470	PZ221494	**This study**
***Helicocollum bambusae***	**SICAUCC 25-0191**	PZ205063	PZ221471	PZ221495	**This study**
*Helicocollum chanthaburiensis*	BCC 80047 T	KT222330	KT222343	–	Luangsa-ard et al. (2017)
*Helicocollum krabiensis*	BCC 68582 T	KT222327	KT222338	KT222331	Luangsa-ard et al. (2017)
*Helicocollum krabiensis*	BCC 68583	KT222326	KT222339	KT222332	Luangsa-ard et al. (2017)
*Helicocollum krabiensis*	BCC 68584	KT222325	KT222340	KT222333	Luangsa-ard et al. (2017)
*Helicocollum samlanense*	BCC 96785 T	PP447259	PP453598	–	Crous et al. (2024)
*Helicocollum samlanense*	BCC 96783	PP447258	PP453597	–	Crous et al. (2024)
*Helicocollum surathaniense*	BCC 34463	KT222328	KT222336	–	Luangsa-ard et al. (2017)
*Helicocollum surathaniense*	BCC 34464 T	KT222329	KT222337	–	Luangsa-ard et al. (2017)
*Hypocrella calendulina*	BCC 20309 T	GU552154	–	–	Mongkolsamrit et al. (2011)
*Hypocrella cf. discoidea*	I93-901D	EU392567	EU392646	EU392700	Chaverri et al. (2008)
*Hypocrella cf. discoidea*	I95-901D	EU392568	EU392647	EU392701	Chaverri et al. (2008)
*** Hypocrella Cinnamomum ***	**SICAUCC 25-0192 T**	PZ205056	PZ221472	PZ221496	**This study**
*** Hypocrella Cinnamomum ***	**SICAUCC 25-0193**	PZ205057	PZ221473	PZ221497	**This study**
*Hypocrella citrina*	P.C. 597	AY986905	AY986930	–	Chaverri et al. (2005b)
*Hypocrella disciformis*	P.C. 655	EU392560	EU392643	EU392697	Chaverri et al. (2008)
*Hypocrella disciformis*	P.C. 676	EU392566	EU392645	EU392699	Chaverri et al. (2008)
*Hypocrella discoidea*	BCC 2097	–	AY986945	DQ000346	Chaverri et al. (2005b)
*Hypocrella hirsute*	P.C. 436.2	AY986922	AY986949	DQ000350	Chaverri et al. (2008)
*Hypocrella hirsute*	P.C. 543 T	EU392569	EU392648	EU392702	Chaverri et al. (2008)
*Hypocrella khonsanitii*	BCC 71371	PQ560535	PQ585793	PQ585795	Crous et al. (2025)
*Hypocrella khonsanitii*	BCC 69112 T	PQ560534	PQ585792	PQ585794	Crous et al. (2025)
*Hypocrella limushanensis*	YHH 2303015 T	OR828401	OR832089	OR837107	Wang et al. (2025b)
*Hypocrella limushanensis*	YHH 2303016	OR828402	OR832090	OR837108	Wang et al. (2025b)
*Hypocrella viridans*	P.C. 635	EU392572	EU392651	EU392705	Chaverri et al. (2008)
*Hypocrella viridans*	P.C. 670	EU392574	EU392652	EU392706	Chaverri et al. (2008)
*Hypocrella yunnanensis*	YHH 2305020 T	OR828417	OR854260	OR837109	Wang et al. (2025b)
*Hypocrella yunnanensis*	YHH 2305021	–	OR854261	OR837110	Wang et al. (2025b)
*Purpureocillium lilacinum*	CBS 284.36 T	FR775484	EF468792	EF468898	Luangsa-ard et al. (2011)
*Purpureocillium lilacinum*	CBS 431.87	EF468844	EF468791	EF468897	Luangsa-ard et al. (2011)
*Regiocrella camerunensis*	ARSEF 7682 T	DQ118735	DQ118743	DQ127234	Chaverri et al. (2005a)
***Regiocrella sichuanensis***	**SICAUCC 25-0188 T**	PZ205075	PZ221474	PZ221498	**This study**
***Regiocrella sichuanensis***	**SICAUCC 25-0189**	PZ205076	PZ221475	PZ221499	**This study**
*Regiocrella sinensis*	CUP CH-2640 T	DQ118736	DQ118744	DQ127235	Chaverri et al. (2005a)

Notes: “T” Type material. “–” means that the sequence is missing or unavailable. Sequences obtained in this study are shown in bold.

## Results

### Molecular phylogeny of *Clavicipitaceae*

**Figure 1.**
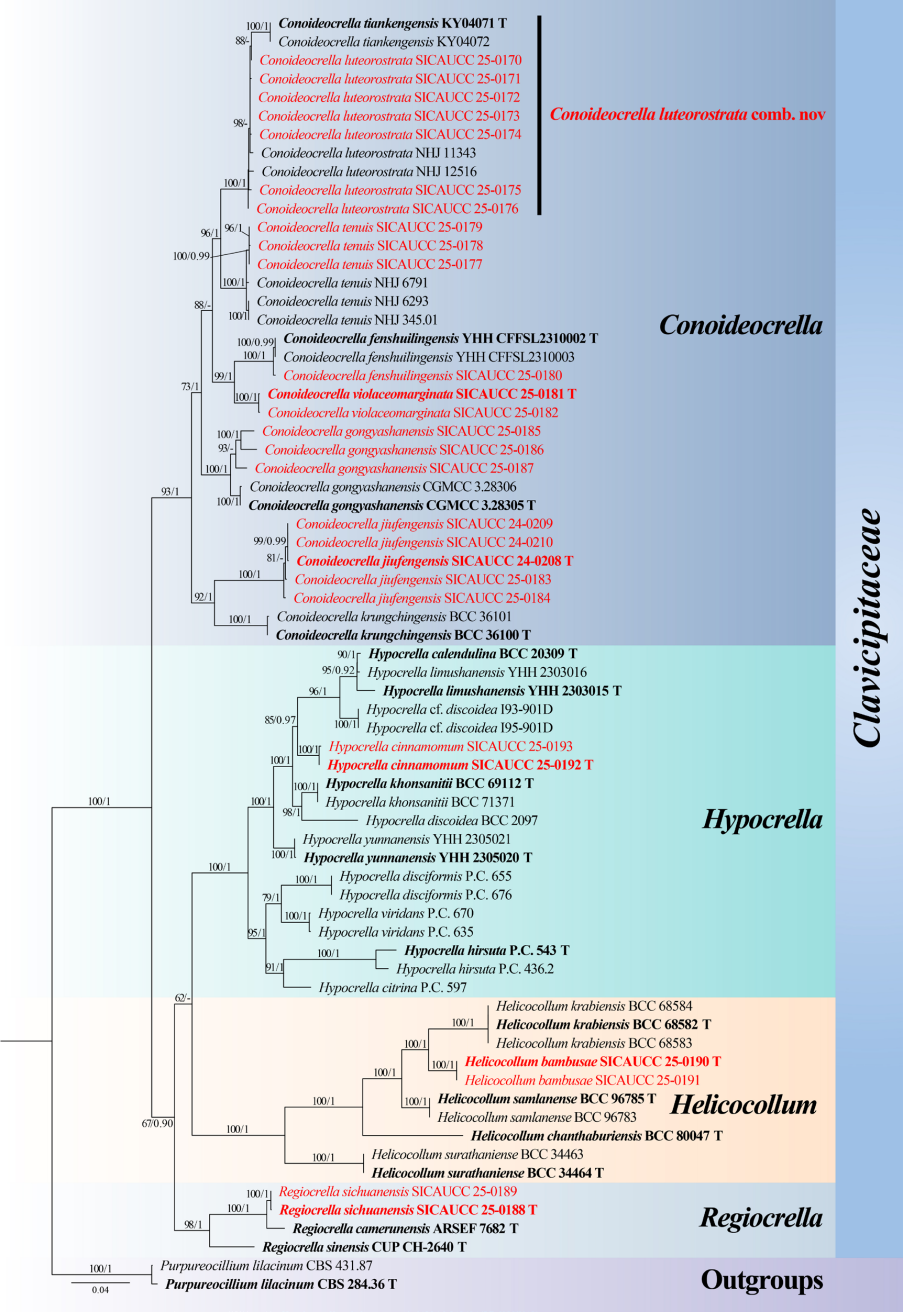
RAxML tree based on the concatenated three gene datasets (LSU, *tef*1-α, and *rpb*1) showing the relationships among *Clavicipitaceae* and related genera. The RAxML bootstrap support values (MLBS) equal to or above 60% and Bayesian posterior probabilities (BPP) equal to or above 0.90 are given at the nodes (MLBS/BPP). The scale bar represents the expected number of changes per site. The ex-type strains are in bold and marked with a postscript “T”. The newly generated sequences in this study are highlighted in red. The tree was rooted to *Purpureocillium lilacinum* (CBS 284.36) and *P. lilacinum* (CBS 431.87).

The concatenated and aligned three-locus dataset (LSU, *tef*1-α, and *rpb*1) was compiled for 69 taxa of *Clavicipitaceae*, with *Purpureocillium lilacinum* CBS 284.36 and *P. lilacinum* CBS 431.87 (*Ophiocordycipitaceae*, *Hypocreales*) designated as outgroup taxa (Fig. 1, Table 1). The final alignment consisted of 2,793 bp (including gaps), with the following partitions: LSU: 981 bp, *tef*1-α: 1,029 bp, and *rpb*1: 783 bp. This alignment matrix comprised 1,167 distinct patterns, with 17.61% undetermined characters or gaps across the dataset. The gamma distribution shape parameter (α) was estimated at 0.213019, and the total tree length was 1.429163. The best-scoring RAxML maximum likelihood (ML) tree yielded a final log-likelihood value of −16715.485444. Phylogenetic topologies inferred from Bayesian inference (BI) were highly congruent with those from ML analysis. Consequently, only the best-scoring RAxML tree inferred from the concatenated dataset was presented in Fig. 1, with ML bootstrap support (MLBS ≥ 60%) and Bayesian posterior probabilities (BPP ≥ 0.90) indicated at each corresponding node. This multilocus phylogeny provided robust support for the recognition of five novel taxa and four new provincial distributional records in *Clavicipitaceae*. *Conoideocrella luteorostrata*, *C. tenuis*, *C. fenshuilingensis*, and *C. gongyashanensis* were reported herein as new geographical records for Sichuan Province. *Conoideocrella jiufengensis*, *C. violaceomarginata*, *Helicocollum bambusae*, *Hypocrella cinnamomum*, and *Regiocrella sichuanensis* were formally recognized as novel taxa, with strong bootstrap supports for their phylogenetic placements.

### Taxonomy

#### Conoideocrella fenshuilingensis

Taxon classificationAnimaliaHypocrealesClavicipitaceae

Hong Yu bis, Zhi Li Yang, Z.Q. Wang & J.M. Ma, J. Fungi 10(6): 13 (2024)

BA2FE631-6A0F-5F27-84CA-2F4EFE3E044B

Fig. 2

##### Description.

Parasitic on scale insects (*Coccidae*, *Hemiptera*) from *Camellia sinensis* (L.) Kuntze (*Theaceae*). ***Teleomorph*: *Stromata*** creamy white to dirty white, entirely covering the scale insects, flattened scutate or hemi-globose, cottony, surrounded by a creamy-white dense hypothallus, 770–900 µm in diameter. Upon subsequent digestion, pale yellow mycelial residues remain. ***Perithecia*** mostly distributed on the hypothallus, or poorly at the margin of the stroma, scattered, pale olive gold, covered with milky white hyphae, elongated flask-shaped or elongated conic shape. ***Anamorph***: hirsutella-like.

**Figure 2.**
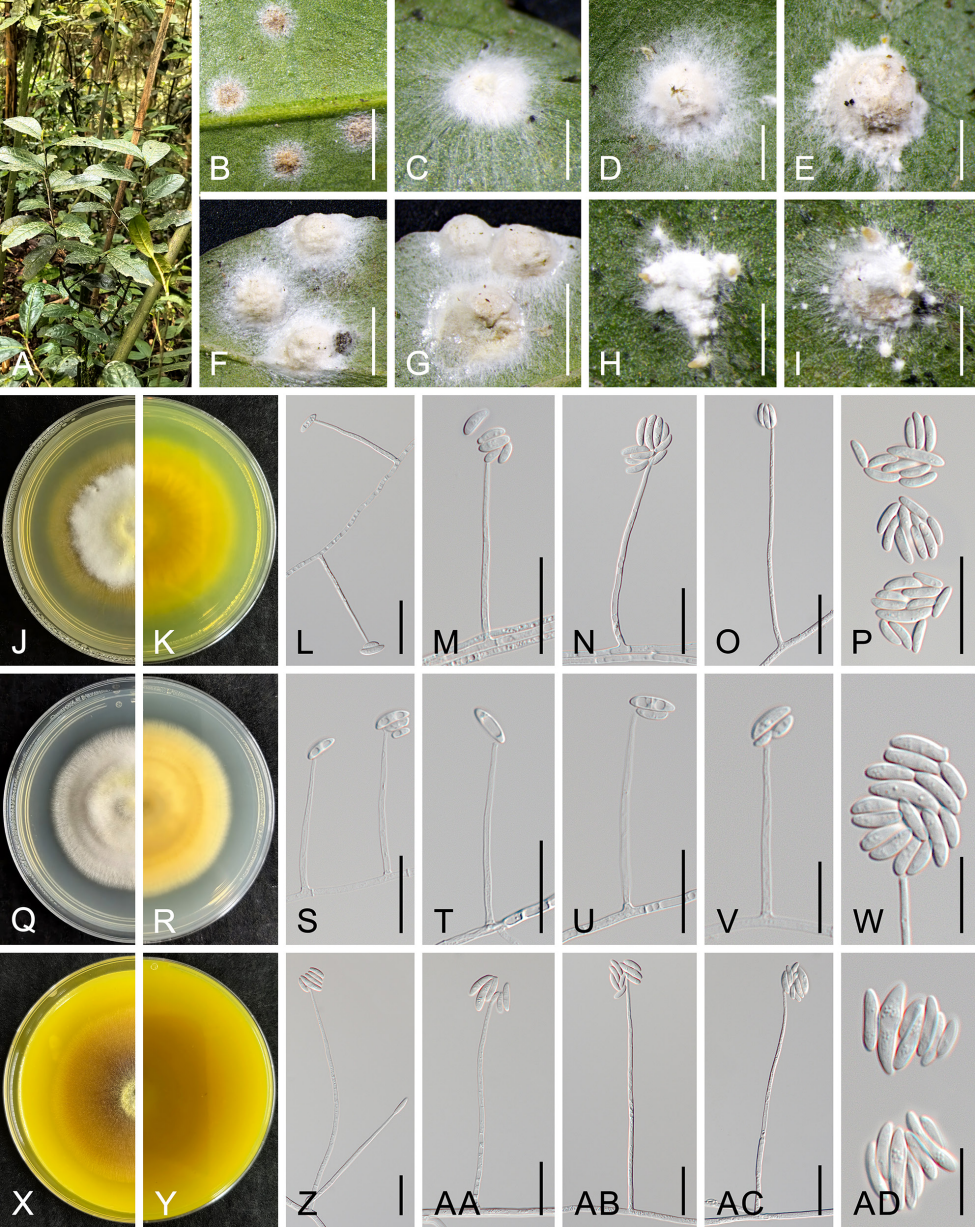
*Conoideocrella fenshuilingensis* (SICAU 25-0212, living culture SICAUCC 25-0180). **A** Habitat; **B–G** Anamorphic stromata containing conidiomata; **H, I** Telemorphic stroma containing perithecia; **J, K** Colonies on PDA after 21 d; **L–P** Conidiophores bearing phialides and conidia on PDA; **Q, R** Colonies on SDAY/4 after 21 d; **S–W** Conidiophores bearing phialides and conidia on SDAY/4; **X, Y** Colonies on OA after 21 d; **Z–AD** Conidiophores bearing phialides and conidia on OA. Scale bars: 1000 µm (**B–G**); 500 µm (**H, I**); 20 µm (**L–P, S–U, Z–AC**); 10 µm (**V, W, AD**).

##### Culture characteristics.

Colonies on PDA reaching 38–42 mm diam after 21 d at 25 °C, pale yellow in the center, creamy white at periphery, circular, flat, floccose, abundant aerial mycelium, without aerial mycelium towards periphery, margin entire, reverse vivid golden yellow, with yellow pigment diffusing into the medium. ***Conidiophores*** solitary, erect, straight or slightly curved, arising directly from aerial hyphae, usually reduced to single phialides, unbranched, 1-septate at base, gradually tapering towards the apex, hyaline, smooth-walled. ***Phialides*** narrowly cylindrical or subulate, lateral, (33–)40–67(–78) × 1.3–2.1 µm. ***Conidia*** fusiform and slightly curved, arranged in slimy heads, 0–1-septate, hyaline, smooth-walled, 5.8–8.5(–9.4) × 1.7–2.4 µm. ***Chlamydospores*** not observed.

Colonies on SDAY/4 reaching 38–40 mm diam after 21 d at 25 °C, off-white, circular, flat, slightly convex in the center, crater-shaped, floccose to cottony, abundant aerial mycelium, generating several concentric rings, margin entire, reverse pale straw yellow. ***Conidiophores*** solitary, erect, straight or slightly curved, arising directly from aerial hyphae, usually reduced to single phialides, unbranched, with a single basal septum, gradually tapering towards the apex, hyaline, smooth-walled. ***Phialides*** narrowly cylindrical or subulate, lateral, (24–)33–38(–46) × 1.3–3 µm. ***Conidia*** fusiform and slightly curved, arranged in slimy heads, 0–1-septate, hyaline, smooth-walled, (5.5–)6.5–8 × 1.9–2.8(–3.2) µm. ***Chlamydospores*** not observed.

Colonies on OA reaching 35–38 mm diam after 21 d at 25 °C, dark umber brown, circular, flat, floccose, sparse aerial mycelium, arranged in radially lines, margin entire, reverse dark bronze brown, with yellow pigment diffusing into the medium. ***Conidiophores*** solitary, erect, straight or slightly curved, arising directly from aerial and substratal hyphae, usually reduced to single phialides, unbranched, with a single basal septum, gradually tapering towards the apex, hyaline, smooth-walled, with cell walls usually thinner than those of vegetative hyphae. ***Phialides*** narrowly cylindrical or subulate, lateral, (50–)60–108(–150) × 1.9–2.4 µm. ***Conidia*** fusiform and slightly curved, arranged in slimy heads, 0–1-septate, hyaline, smooth-walled, (6.7–)7.5–13(–14.4) × 1.9–3.1 µm. ***Chlamydospores*** not observed.

##### Hosts.

Scale insect (*Coccidae*, *Hemiptera*).

##### Known distribution.

China (Yunnan, Sichuan).

##### Material examined.

CHINA • Sichuan Province, Meishan City, Hongya County, Gaomiao Town, Huayuan Village, 29°35'34"N, 103°14'07"E, on scale insects on the abaxial surface of living leaves of *Camellia sinensis*, 18 Mar. 2025, Feng Liu, SICAU 25-0212, living culture SICAUCC 25-0180.

##### Notes.

*Conoideocrella fenshuilingensis* was originally isolated from scale insects in Yunnan Province, with only the morphological features of its teleomorph having been described (Wang et al. 2024b). In the present phylogenetic analysis, strain SICAUCC 25-0180 clustered within the clade of *C. fenshuilingensis* (culture ex-type YHH CFFSL2310002 and YHH CFFSL2310003) with maximal statistical support (100% MLBS/1.00 BPP; Fig. 1). Morphologically, our strain exhibits stromatal and perithecial characteristics comparable to those of the type specimen, with the only discrepancy being perithecial color (pale olive gold vs. pale brown to black) (Wang et al. 2024b). The color variation may be attributable to a different developmental stage or environmental factors. This study constitutes the first report of *C. fenshuilingensis* in Sichuan Province, additionally documents the first simultaneous observation of both anamorphic and teleomorphic states for the taxon.

#### Conoideocrella gongyashanensis

Taxon classificationAnimaliaHypocrealesClavicipitaceae

L.B. Lin & J.Zhi Qiu, Frontiers Microbiol. 16: 11 (2025)

F453B7B4-6511-5138-B39E-908333840482

Fig. 3

##### Description.

Parasitic on scale insects (*Coccidae*, *Hemiptera*) from *Chimonobambusa* Makino. ***Teleomorph*: *Stromata*** bright yellow to yellowish brown, entirely covering the scale insects, pulvinate or scutate, cottony, surrounded by a yellowish brown dense hypothallus. ***Perithecia*** densely distributed on the hypothallus, a few at the margin of the stroma, clustered, slightly convex, basal bright yellow, topmost bright orange, elongated flask-shaped to ovoid, ostiolate with long-beaked papilla. ***Anamorph***: hirsutella-like.

**Figure 3.**
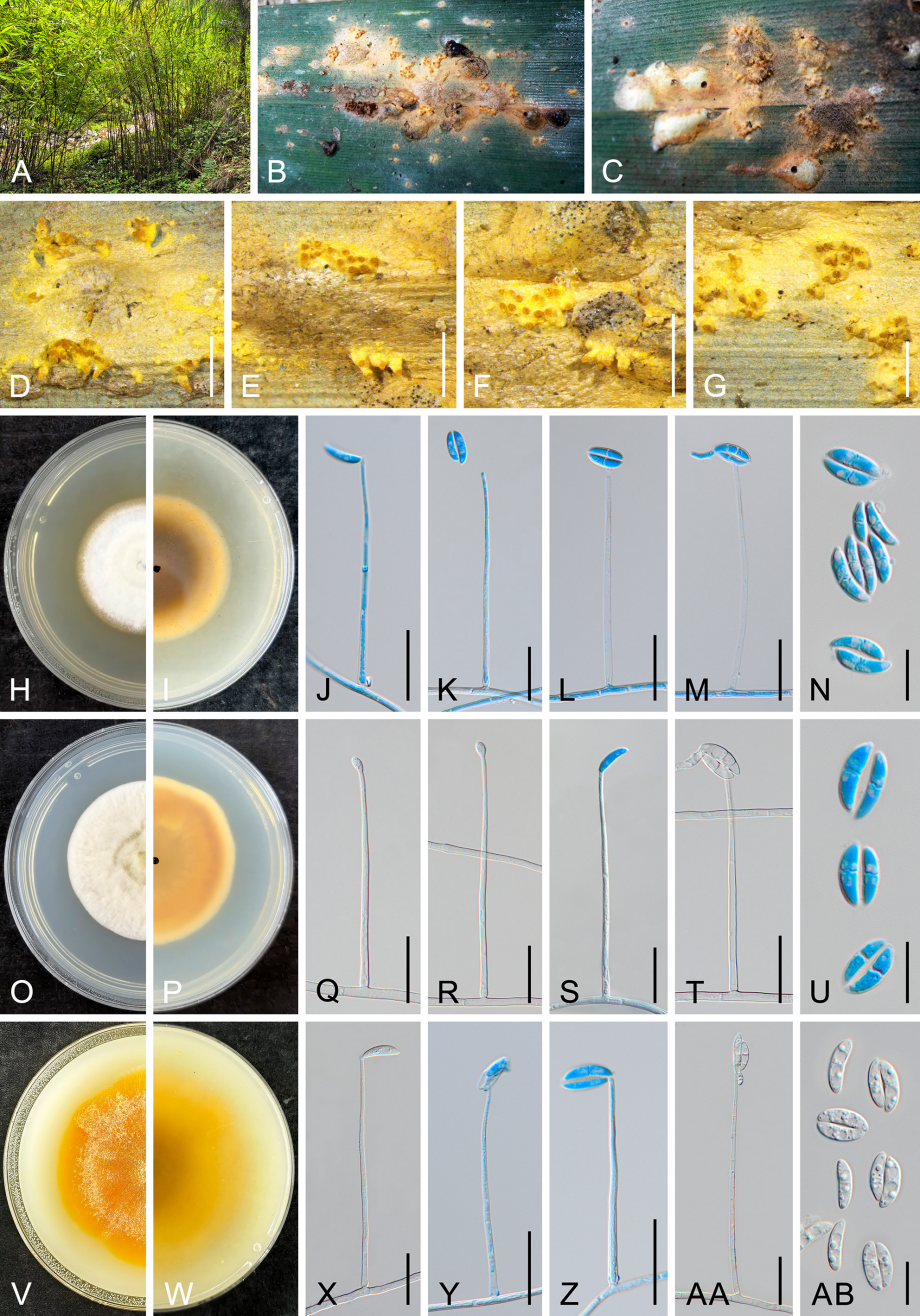
*Conoideocrella gongyashanensis* (SICAU 25-0217, living culture SICAUCC 25-0185). **A** Habitat; **B, C** Fungus on the hosts; **D–G** Telemorphic stromata containing perithecia; **H, I** Colonies on PDA after 21 d; **J–N** Conidiophores bearing phialides and conidia on PDA; **O, P** Colonies on SDAY/4 after 21 d; **Q–U** Conidiophores bearing phialides and conidia on SDAY/4; **V, W** Colonies on OA after 21 d; **X–AB** Conidiophores bearing phialides and conidia on OA. Scale bars: 1000 µm (**D–G**); 20 µm (**J–M, Q–T, X–AA**); 10 µm (**N, U, AB**).

##### Culture characteristics.

Colonies on PDA reaching 27–30 mm diam after 21 d at 25 °C, cream white, circular, flat, felty, abundant aerial mycelium, arranged radially, generating several concentric rings, closely appressed to the agar surface, margin entire, reverse central dark brown, pale olive gold at periphery. ***Conidiophores*** solitary, erect, straight or slightly curved, arising directly from aerial hyphae, monophialidic, unbranched, with a single basal septum, gradually tapering from the base to the apex, hyaline, smooth-walled, with cell walls usually thinner than those of vegetative hyphae. ***Phialides*** narrowly cylindrical, acicular or subulate, lateral, 60–80(–90) × 2–2.8 µm. ***Conidia*** fusiform and slightly curved, producing 2 conidia per phialide, 1–2-septate, hyaline, smooth-walled, with large and small guttules, 10–15 × 2.3–3.7 µm. ***Chlamydospores*** not observed.

Colonies on SDAY/4 reaching 30–32 mm diam after 21 d at 25 °C, cream white, circular, flat, floccose or felty, crater-shaped, abundant aerial mycelium, closely appressed to the agar surface, margin entire, reverse uniformly pale golden beige. ***Conidiophores*** solitary, erect, straight or slightly curved, straight, arising directly from aerial hyphae, monophialidic, unbranched, with a single basal septum, gradually tapering from the base to the apex, hyaline, smooth-walled, with cell walls usually thinner than those of vegetative hyphae. ***Phialides*** narrowly cylindrical or subulate, lateral, (54–)65–80(–85) × 2.1–3.4 µm. ***Conidia*** fusiform and slightly curved, producing 2 conidia per phialide, 1–2-septate, hyaline, smooth-walled, with large and small guttules, 9–14.5 × 2.6–4.2 μm. ***Chlamydospores*** not observed.

Colonies on OA reaching 40–42 mm diam after 21 d at 25 °C, bright golden orange to dark apricot brown, circular, flat, membranous, sparse aerial mycelium, arranged in radial lines, closely appressed to the agar surface, margin entire, reverse pale orange yellow. ***Conidiophores*** solitary, erect, straight or slightly curved, arising directly from aerial hyphae, monophialidic, unbranched, with a single basal septum, gradually tapering from the base to the apex, hyaline, smooth-walled, with cell walls usually thicker than those of vegetative hyphae. ***Phialides*** narrowly cylindrical or subulate, lateral, 70–85(–98) × 2.1–3.4 µm. ***Conidia*** fusiform and slightly curved, producing 2 conidia per phialide, 1–2-septate, hyaline, smooth-walled, with large and small guttules, 10–14(–16) × 2.3–3.5(–4.3) µm. ***Chlamydospores*** not observed.

##### Hosts.

Spiders (*Araneae*), scale insects (*Coccidae*, *Hemiptera*).

##### Known distribution.

China (Fujian, Sichuan).

##### Material examined.

CHINA • Sichuan Province, Ya’an City, Yingjing County, Longcanggou Town, Longcanggou National Forest Park, 29°38'39"N, 102°52'47"E, on scale insects on the abaxial surface of living leaves of *Chimonobambusa* Makino, 18 Jul. 2025, Feng Liu, SICAU 25-0217, living culture SICAUCC 25-0185; • ibid. SICAU 25-0218, living culture SICAUCC 25-0186; Meishan City, Hongya County, Gaomiao Town, Huayuan Village, 29°35'34"N, 103°14'07"E, on scale insects on the abaxial surface of living leaves of *Camellia sinensis*, 17 Mar. 2025, Feng Liu, SICAU 25-0219, living culture SICAUCC 25-0187.

##### Notes.

In our phylogenetic analysis, our new isolates (SICAUCC 25-0185 to 25-0187) clustered with *Conoideocrella gongyashanensis* (culture ex-type CGMCC 3.28305 and CGMCC 3.28306) with 100% MLBS and 1.00 BPP statistical support (Fig. 1). Morphologically, our new isolates are similar to the ex-type strain of *C. gongyashanensis* in possessing monophialidic, unbranched, septate, hyaline conidiophores, lateral, cylindrical or subulate phialides, and fusiform, guttulate, 1–2-septate, hyaline conidia. Based on phylogenetic analysis and morphological characteristics, we identified our new isolates as *C. gongyashanensis*. Lin et al. (2025) found *C. gongyashanensis* parasitizing dead spiders attached to fallen leaves in Fujian Province, and originally described the morphological features of its anamorph. In contrast, our new isolates were collected from scale insects on the abaxial surface of living leaves of *Chimonobambusa* Makino and *Camellia sinensis* (L.) Kuntze in Sichuan Province, and this study documents the first simultaneous observation of both anamorphic and teleomorphic states for this taxon. This identification represents a new host and a new geographical record for this species in Sichuan Province, China.

#### Conoideocrella jiufengensis

Taxon classificationAnimaliaHypocrealesClavicipitaceae

Feng Liu & Chun L. Yang sp. nov.

7734B4CF-9F62-5249-9663-35B296926B0F

Index Fungorum: IF903433

Fig. 4

##### Etymology.

Name refers to Jiufeng Mountain where it was collected.

##### Diagnosis.

Similar to *Conoideocrella krungchingensis* in having comparable morphology of stromata, conidiophores, and conidia, but *C. jiufengensis* differs by its larger stromata with different color, longer phialides, transverse septa of conidia, and significant nucleotide differences in LSU and *tef*1-α.

##### Type.

CHINA • Sichuan Province, Pengzhou City, Jiufeng Mountain, 31°18'50"N, 103°51'17"E, on scale insects from *Chimonobambusa quadrangularis* (Franceschi) Makino, 11 Dec. 2023, Feng Liu, **holotype**SICAU 24-0194, culture ex-type SICAUCC 24-0208.

##### Description.

Parasitic on the scale insects (*Coccidae*, *Hemiptera*) attached to the underside of living leaves of bamboo. ***Teleomorph***: Infected hosts were covered with orange yellow to pure yellow mycelium layer. ***Stromata*** pale yellow to orange yellow, entirely covering and arising from the hosts, pulvinate, cottony, surrounded by orange yellow to pure yellow hypothallus. ***Perithecia*** mostly distributed on the hypothallus, or poorly at the margin of the stroma, scattered or clustered, visible as raised, basal pure yellow, topmost orange yellow, elongated flask-shaped to ovoid, 385–500 × 150–240 µm, ostiolate with long beaked papilla. ***Anamorph***: hirsutella-like.

**Figure 4.**
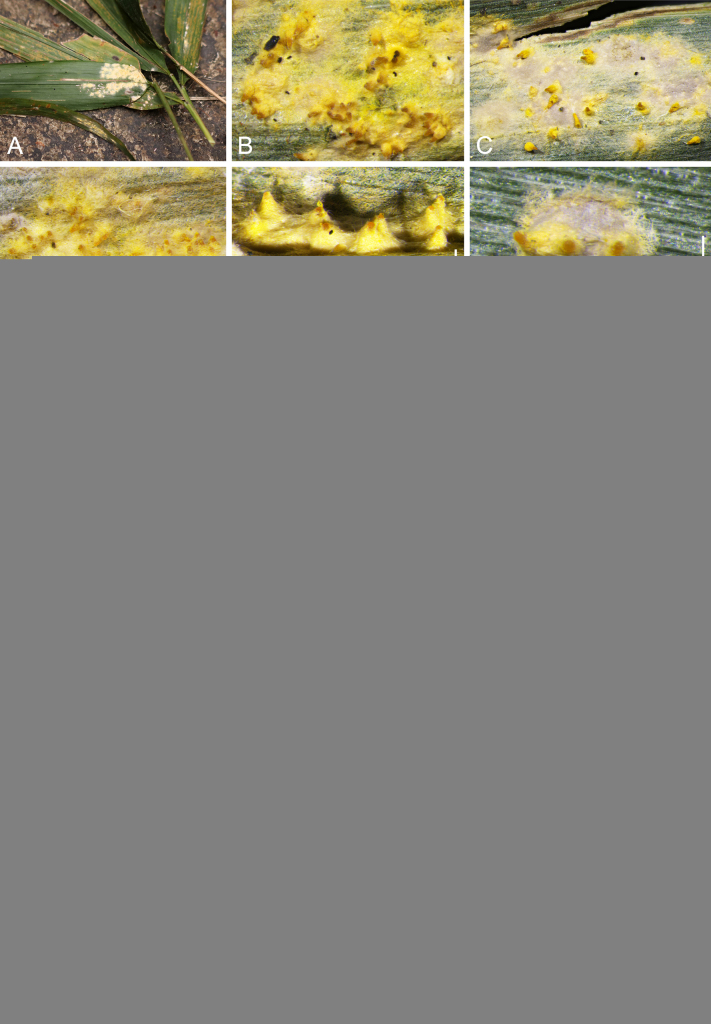
*Conoideocrella jiufengensis* (holotype SICAU 24-0194, culture ex-type SICAUCC 24-0208). **A–C** Fungus on the hosts; **D–F** Telemorphic stromata containing perithecia; **G, H** Colonies on PDA after 21 d; **I–M** Conidiophores bearing phialides and conidia on PDA; **N, O** Colonies on SDAY/4 after 21 d; **P–T** Conidiophores bearing phialides, conidia, and chlamydospores on SDAY/4; **U, V** Colonies on OA after 21 d; **W–AA** Conidiophores bearing phialides and conidia on OA. Scale bars: 1000 µm (**D**); 500 µm (**E, F**); 10 µm (**I–M, P–T, W–AA**).

##### Culture characteristics.

Colonies on PDA reaching 28–30 mm diam after 21 d at 25 °C, pale brown in the center, orange yellow at periphery, circular, flat, cottony to felty with abundant aerial mycelium, closely appressed to the agar surface, radially folded, margin entire, reverse rust. ***Conidiophores*** solitary, erect, straight, arising directly from aerial and substratal mycelium, usually reduced to single phialides, unbranched, with a single basal septum, gradually tapering from the base to the apex, hyaline, smooth-walled, with cell walls usually thinner than those of vegetative hyphae. ***Phialides*** cylindrical or subulate, lateral, (48.7–)50–80(–88.5) × 1.6–3.1 µm. ***Conidia*** fusiform and slightly curved, arranged in a group of two at the neck apex, 1-septate, hyaline, smooth-walled, (9.5–)10–16.2 × 2–3.6 µm. ***Chlamydospores*** not observed.

Colonies on SDAY/4 reaching 25–28 mm diam after 21 d at 25 °C, pale yellow to greyish red, circular, flat, cottony to felty with abundant aerial mycelium, closely appressed to the agar surface, slightly radially folded, margin entire, reverse rust in the center and pale yellow at the margin. ***Conidiophores*** solitary, erect, straight, arising directly from aerial and substratal mycelium, usually reduced to single phialides, unbranched, with a single basal septum, gradually tapering from the base to the apex, hyaline, smooth-walled, with cell walls usually thinner than those of vegetative hyphae. ***Phialides*** cylindrical or subulate, lateral, (68.7–)70–90(–95.5) × 1.7–3.1 µm. ***Conidia*** fusiform and slightly curved, arranged in a group of two at the neck apex, 1-septate, hyaline, smooth-walled, 10–16.1 × 2.5–3.6(–4.1) µm. ***Chlamydospores*** present, terminal or lateral, cylindrical or hockey-stick shaped, flexuous, 1–3 septate, hyaline, smooth-, thick-walled.

Colonies on OA reaching 28–30 mm diam after 21 d at 25 °C, greyish white to orange yellow, circular, flat, floccose with moderate aerial mycelium, arranged in radially lines, closely appressed to the agar surface, margin entire, reverse pale orange yellow. ***Conidiophores*** solitary, erect, straight, arising directly from aerial and substratal mycelium, usually reduced to single phialides, unbranched, with a single basal septum, gradually tapering from the base to the apex, hyaline, smooth-walled, with cell walls usually thinner than those of vegetative hyphae. ***Phialides*** cylindrical or subulate, lateral, (56.6–)60–80(–92) × 1.6–2.7 µm. ***Conidia*** fusiform and slightly curved, arranged in a group of two at the neck apex, 1-septate, hyaline, smooth-walled, 7.2–15 × 2.1–3.6 µm. ***Chlamydospores*** not observed.

##### Hosts.

Scale insects (*Coccidae*, *Hemiptera*).

##### Known distribution.

China (Sichuan).

##### Additional specimen examined.

CHINA • Sichuan Province, Pengzhou City, Jiufeng Mountain, 31°18'50"N, 103°51'17"E, on scale insects from *Chimonobambusa quadrangularis* (Franceschi) Makino, 11 Dec. 2023, Feng Liu, living culture SICAUCC 24-0209; • ibid. living culture SICAUCC 24-0210; Ya’an City, Yingjing County, Longcanggou Town, Longcanggou National Forest Park, 29°38'39.59"N, 102°52'47.99"E, on scale insects on the abaxial surface of living leaves of *Chimonobambusa* Makino, 18 Jul. 2025, Feng Liu, SICAU 25-0215, living culture SICAUCC 25-0183; Ya’an City, Yingjing County, Chadi Slope, 29°48'38"N, 102°55'45"E, on scale insects infesting the stalks of *Chimonobambusa purpurea* J. R. Xue & T. P. Yi, 14 Mar. 2025, Feng Liu, SICAU 25-0216, living culture SICAUCC 25-0184.

##### Notes.

In our multi-gene phylogenetic analysis (Fig. 1), *Conoideocrella jiufengensis* (SICAUCC 24-0208 to 24-0210) formed a well-supported sister clade to *C. krungchingensis. Conoideocrella jiufengensis* produces larger stromata (385–500 × 150–240 µm vs. 220–420 × 100–180 µm) and differs in stromatal color (bright yellow to orange-yellow vs. pale yellow, orange to reddish-brown) compared with *C. krungchingensis* (Mongkolsamrit et al. 2016). Conidiophores and conidia of the two species are morphologically similar. However, *C. jiufengensis* differs significantly from *C. krungchingensis* in producing longer phialides (50–80 × 1.6–3.1 µm vs. 30–40 × 1–2 µm), and conidia with fewer transverse septa (1-septate vs. 3–4 septate) on PDA (Mongkolsamrit et al. 2016). The nucleotide differences between our isolate (SICAUCC 24-0208) and the ex-type strain of *C. krungchingensis* (BCC 36100) are 32.38% (18/715, 0 gaps) and 7.37% (66/895, 0 gaps) in the LSU and *tef*1-α. Consequently, based on combined molecular and morphological evidence, we propose *C. jiufengensis* as a novel species.

#### Conoideocrella luteorostrata

Taxon classificationAnimaliaHypocrealesClavicipitaceae

(Zimm.) D. Johnson, G.H. Sung, Hywel-Jones & Spatafora, Mycol. Res. 113(3): 286 (2009)

585E0FCD-8441-5196-837D-D6B6EC266BE4

Fig. 5

##### Synonym.

*Conoideocrella tiankengensis* Wan H. Chen, Y.F. Han, J.D. Liang & Jie H. Zhao, MycoKeys 123: 333 (2025)

##### Description.

Parasitic on *Aleurocanthus spiniferus* Quaintance (*Aleyrodidae*, *Hemiptera*) from *Camellia sinensis* (L.) Kuntze (*Theaceae*). ***Teleomorph*: *Stromata*** milky white when immature, becoming dirty white to pale yellow at maturity, entirely covering the insect hosts, thickened pulvinate, scutate or hemi-globose, cottony, surrounded by a snow-white dense hypothallus, 450–1050 µm in diameter. ***Perithecia*** densely distributed at the margin of the stroma or on the hypothallus, clustered, visible as raised, basal pure brown, topmost dark brown, elongated flask-shaped to ovoid, ostiolate with long beaked papilla. ***Anamorph***: hirsutella-like.

**Figure 5.**
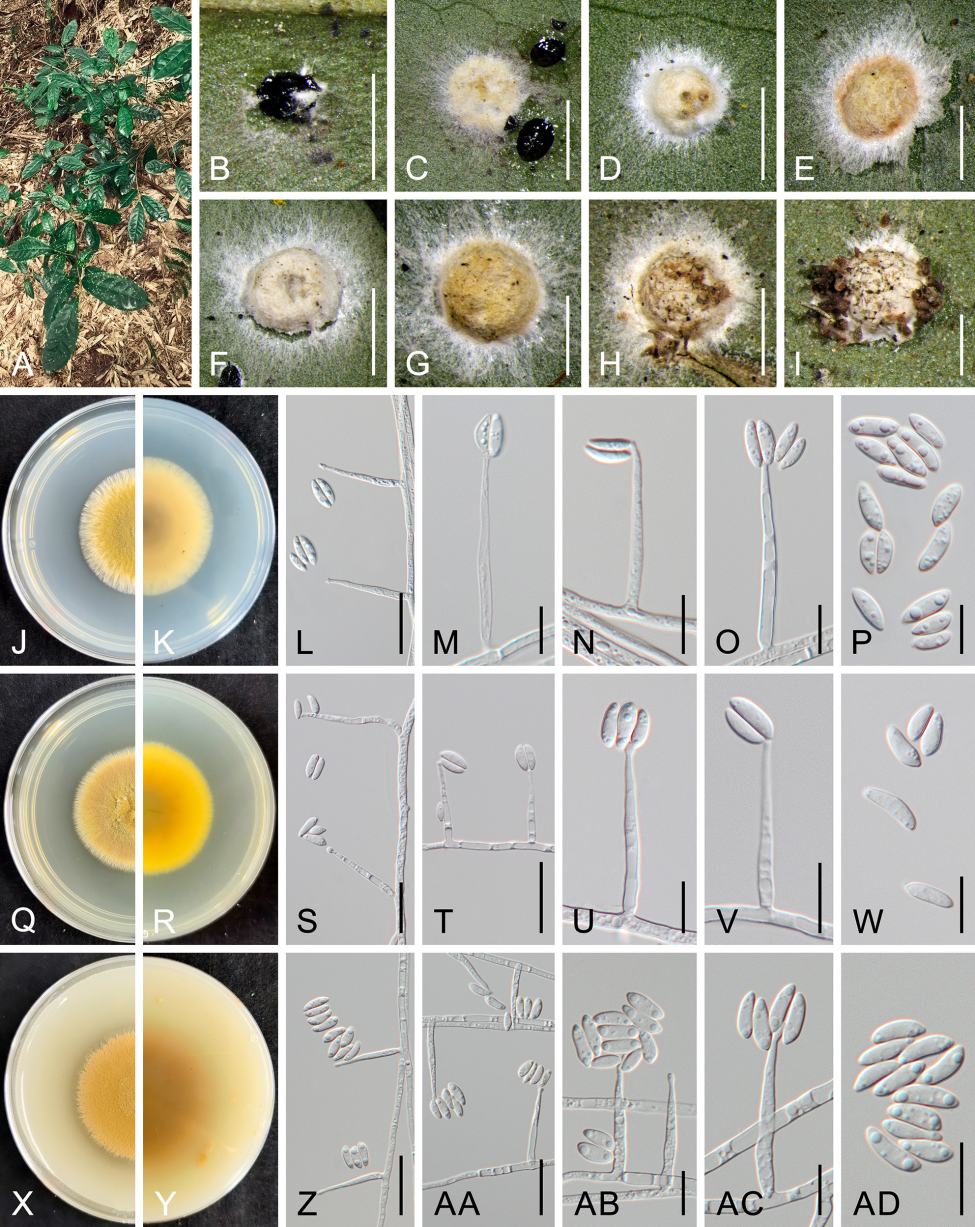
*Conoideocrella luteorostrata* (SICAU 25-0204, living culture SICAUCC 25-0172). **A** Habitat; **B–G** Anamorphic stroma containing conidiomata; **H, I** Telemorphic stroma containing perithecia; **J, K** Colonies on PDA after 21 d; **L–P** Conidiophores bearing phialides and conidia on PDA; **Q, R** Colonies on SDAY/4 after 21 d; **S–W** Conidiophores bearing phialides and conidia on SDAY/4; **X, Y** Colonies on OA after 21 d; **Z–AD** Conidiophores bearing phialides and conidia on OA. Scale bars: 500 µm (**B–I**); 20 µm (**L, S, T, Z, AA**); 10 µm (**M–P, U–W, AB–AD**).

##### Culture characteristics.

Colonies on PDA reaching 27–30 mm diam after 21 d at 25 °C, central greyish green, light-colored at periphery, circular, flat, felty, aerial mycelium moderate, arranged radially, closely appressed to the agar surface, margin entire, reverse pale yellow. ***Conidiophores*** solitary, erect, straight, arising directly from submerged or superficial hyphae, monophialidic, unbranched, with a single basal septum, gradually tapering from the base to the apex, hyaline, smooth-walled, with cell walls usually thinner than those of vegetative hyphae. ***Phialides*** cylindrical, acicular or subulate, lateral, (24–)28–44(–48) × 2–3.4 µm. ***Conidia*** fusiform and slightly curved, arranged in a group of 2–4 at the neck apex, aseptate, hyaline, smooth-walled, with large and small guttules, (6.8–)7.5–11 × 2.5–4 µm. ***Chlamydospores*** not observed.

Colonies on SDAY/4 reaching 30–32 mm diam after 21 d at 25 °C, brown yellow to brass yellow, circular, flat, felty, aerial mycelium moderate, arranged radially, slightly folded, closely appressed to the agar surface, powdery while sporulating, margin entire, reverse dark brown in the center and pale yellow at the margin. ***Conidiophores*** solitary, erect, straight or slightly curved, straight, arising directly from aerial or substratal hyphae, monophialidic, unbranched, with a single basal septum, gradually tapering from the base to the apex, hyaline, smooth-walled, with cell walls usually thinner than those of vegetative hyphae. ***Phialides*** cylindrical or subulate, lateral, (20.7–)24–40(–45.2) × 2.1–3.3 µm. ***Conidia*** fusiform and slightly curved, arranged in a group of 2–3 at the neck apex, aseptate, hyaline, smooth-walled, with large and small guttules, (6.6–)7.2–9.6(–10.3) × 2.5–4 µm. ***Chlamydospores*** not observed.

Colonies on OA reaching 30–32 mm diam after 21 d at 25 °C, brown-yellow to brass yellow, circular, flat, felty, aerial mycelium sparse, arranged in radial lines, closely appressed to the agar surface, intense sporulation, margin entire, reverse pale orange yellow. ***Conidiophores*** solitary, erect, straight, arising directly from aerial or substratal hyphae, monophialidic, unbranched, with a single basal septum, gradually tapering from the base to the apex, hyaline, smooth-walled, with cell walls usually thicker than those of vegetative hyphae. ***Phialides*** cylindrical or subulate, lateral, (16.6–)21–30(–32) × 2–3.1 µm. ***Conidia*** fusiform and slightly curved, arranged in a group of 2–5 at the neck apex, aseptate, hyaline, smooth-walled, with large and small guttules, 7.2–10(–10.7) × 2.2–3.3 µm. ***Chlamydospores*** not observed.

##### Hosts.

Whiteflies (*Aleyrodidae*, *Hemiptera*), scale insects (*Coccidae*, *Hemiptera*).

##### Known distribution.

China (Sichuan), Cuba, Fiji, Ghana, Guatemala, Java, Mexico, New Zealand, Portugal, Samoa, Seychelles, Sri Lanka, Thailand, USA.

##### Material examined.

CHINA • Sichuan Province, Meishan City, Hongya County, Gaomiao Town, Huayuan Village, 29°37'24"N, 103°11'57"E, on *Aleurocanthus spiniferus* on the abaxial surface of living leaves of *Camellia sinensis*, 18 Mar. 2025, Feng Liu, SICAU 25-0204, living culture SICAUCC 25-0172; • ibid. 29°35'34"N, 103°14'07"E, on scale insects on the abaxial surface of living leaves of *Eurya loquaiana* Dunn, 18 Mar. 2025, Feng Liu, SICAU 25-0203, living culture SICAUCC 25-0171; • ibid. SICAU 25-0205, living culture SICAUCC 25-0173; Ya’an City, Yingjing County, Xinghe County, Niubei Mountain Town, Nanmu Village, 29°47'14"N, 102°31'45"E, on scale insects on the abaxial surface of living leaves of *Camellia sinensis*, 19 Jul. 2025, Feng Liu, SICAU 25-0202, living culture SICAUCC 25-0170; Ya’an City, Yingjing County, Longcanggou Town, Yuquan Village, 29°37'53"N, 102°45'56"E, on scale insects on the abaxial surface of living leaves of *Eurya loquaiana*, 5 Sep. 2025, Feng Liu, SICAU 25-0206, living culture SICAUCC 25-0174; Ya’an City, Tianquan County, Xiaohe Town, 30°06'34"N, 102°44'17"E, on scale insects on the abaxial surface of living leaves of *Camellia sinensis*, 25 Sep. 2025, Feng Liu, SICAU 25-0207, living culture SICAUCC 25-0175; Leshan City, Jiajiang County, Huatou Town, Maliu Township, Guankou Village, 29°43'52"N, 103°20'45"E, on scale insects on the abaxial surface of living leaves of *Machilus pauhoi* Kaneh., 19 Mar. 2025, Feng Liu, SICAU 25-0208, living culture SICAUCC 25-0176.

##### Notes.

In the phylogenetic analysis, our isolates (SICAUCC 25-0170 to 25-0176) clustered with *Conoideocrella luteorostrata* (NHJ 11343 and NHJ 12516) and *C. tiankengensis* (KY04071 and KY04072) with 100% MLBS and 1.00 BPP statistical support (Fig. 1). *Conoideocrella tiankengensis* was isolated as an entomopathogenic fungus from scale insects on leaves in karst habitats in Guizhou Province, China (Chen et al. 2025a). Phylogenetically, *C. tiankengensis* is indistinguishable from *C. luteorostrata* (Fig. 1), and base pair comparisons between *C. tiankengensis* (KY04071) and *C. luteorostrata* (NHJ 11343) show 0.12% (1/840 bp, 1 gap) in LSU and 0.32% (3/952 bp, without gaps) in *tef*1-α. Morphologically, *C. tiankengensis* resembles *C. luteorostrata*, although *C. tiankengensis* has shorter conidiophores (13.6–23.2 × 1.6–2.6 μm vs. 150–240 × 2.0–3.0 μm) (Hywel-Jones 1993; Chen et al. 2025a). Our isolates resemble the specimens of *C. luteorostrata* identified by Hywel-Jones (1993) and Lovett et al. (2022) in terms of morphology and dimensions. Consequently, based on morphological and phylogenetic analyses, *C. tiankengensis* should be regarded as a synonym of *C. luteorostrata*, and our collections are identified as *C. luteorostrata*, representing a new provincial record for Sichuan Province, China.

#### Conoideocrella tenuis

Taxon classificationAnimaliaHypocrealesClavicipitaceae

(Petch) D. Johnson, G.H. Sung, Hywel-Jones & Spatafora, Mycol. Res. 113(3): 286 (2009)

8175F7FC-1EA2-5469-9905-8300B76E936F

Figs 6, 7

##### Description.

Parasitic on scale insects (*Coccidae*, *Hemiptera*) from *Camellia sinensis* (L.) Kuntze (*Theaceae*). ***Teleomorph*: *Stromata*** milky white when young, turning dirty white at maturity, entirely covering the insect hosts, flattened pulvinate or hemi-globose, cottony, surrounded by a creamy-white sparse hypothallus, 1.5–2.8 mm in diameter. ***Perithecia*** densely distributed at the margin of the stroma or a few on the hypothallus, scattered or clustered, pale green when immature, becoming milky white to pale brown at maturity, color darkening from base to apex, elongated flask-shaped or elongated conic shape, ostiolate with long beaked papilla, 495–600(–695) × 170–235 µm. ***Asci*** cylindrical, 8-spored, with thickened ascus apex, hyaline, (234–)275–370(–395) × 4–6.6 µm. ***Ascus cap*** hemispherical, hyaline, 2.2–3.1 × 3–3.4 µm. ***Ascospores*** filiform, hyaline, easily divided into part-spores after maturity, part-spores cylindrical. ***Anamorph***: hirsutella-like.

**Figure 6.**
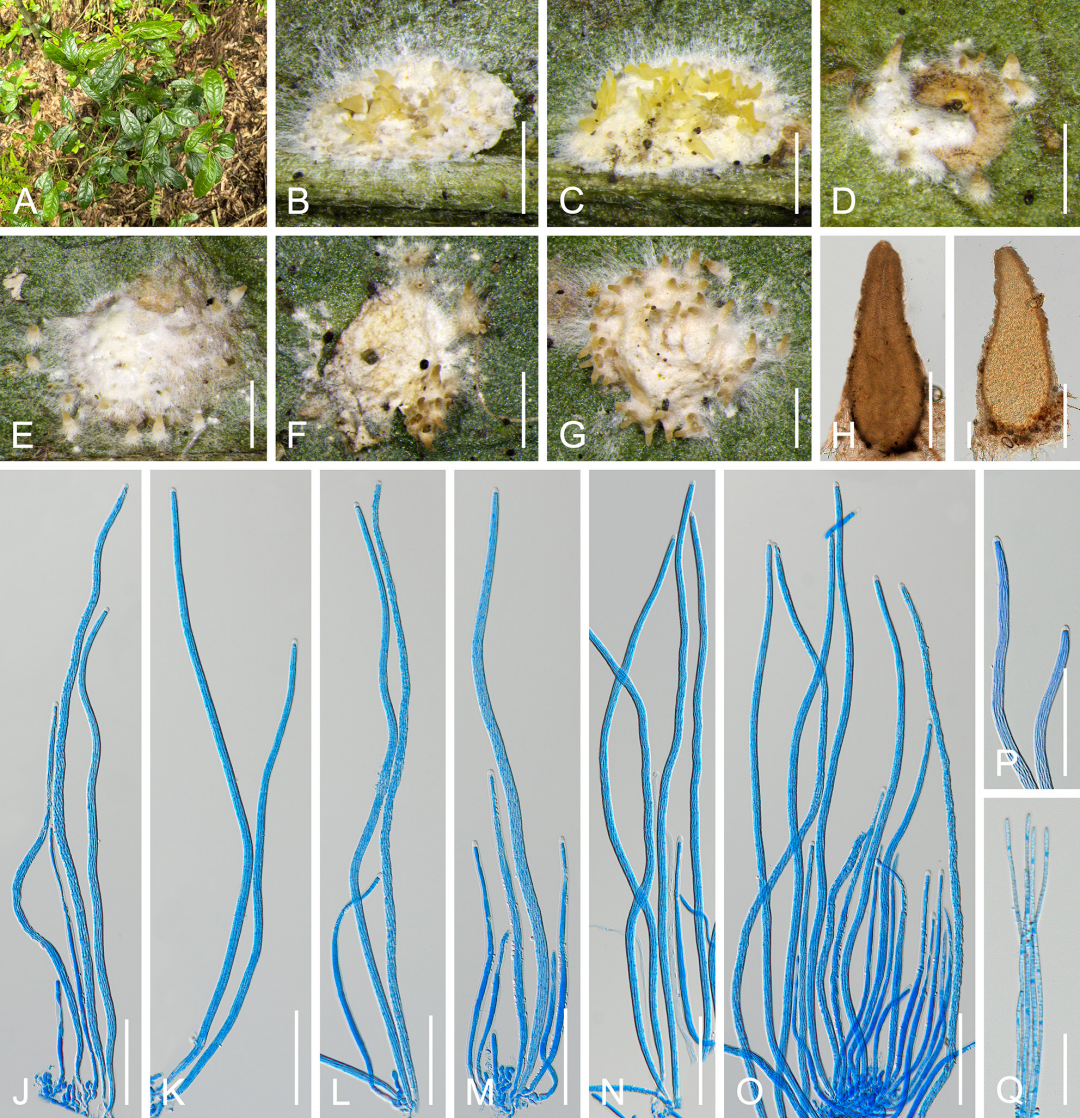
*Conoideocrella tenuis* (SICAU 25-0210). **A** Habitat; **B–G** Telemorphic stroma containing perithecia; **H, I** Perithecium; **J–O** Asci; **P** Apical cap; **Q** Part-spores. Scale bars: 1000 µm (**B–G**); 500 µm (**H, I**); 50 µm (**J–P**); 20 µm (**Q**).

##### Culture characteristics.

Colonies on PDA reaching 25–28 mm diam after 21 d at 25 °C, grayish white, circular, flat, cottony to felty with moderate aerial mycelium, closely appressed to the agar surface, radially folded, margin entire, reverse pale yellow. ***Conidiophores*** solitary, erect, straight, arising directly from aerial and substratal mycelium, usually reduced to single phialides, unbranched, with 1–2 transverse septa, gradually tapering from the base to the apex, hyaline, smooth-walled, with cell walls usually thinner than those of vegetative hyphae. ***Phialides*** slender cylindrical or rod-shaped, lateral, (64–)80–122(–135) × 1.7–2.4 µm. ***Conidia*** fusiform and slightly curved, arranged in a group of 5–10 at the neck apex, aseptate, hyaline, smooth-walled, with guttules, (5.8–)6.5–10.5 × 2.2–3.2(–3.7) µm. ***Chlamydospores*** not observed.

**Figure 7.**
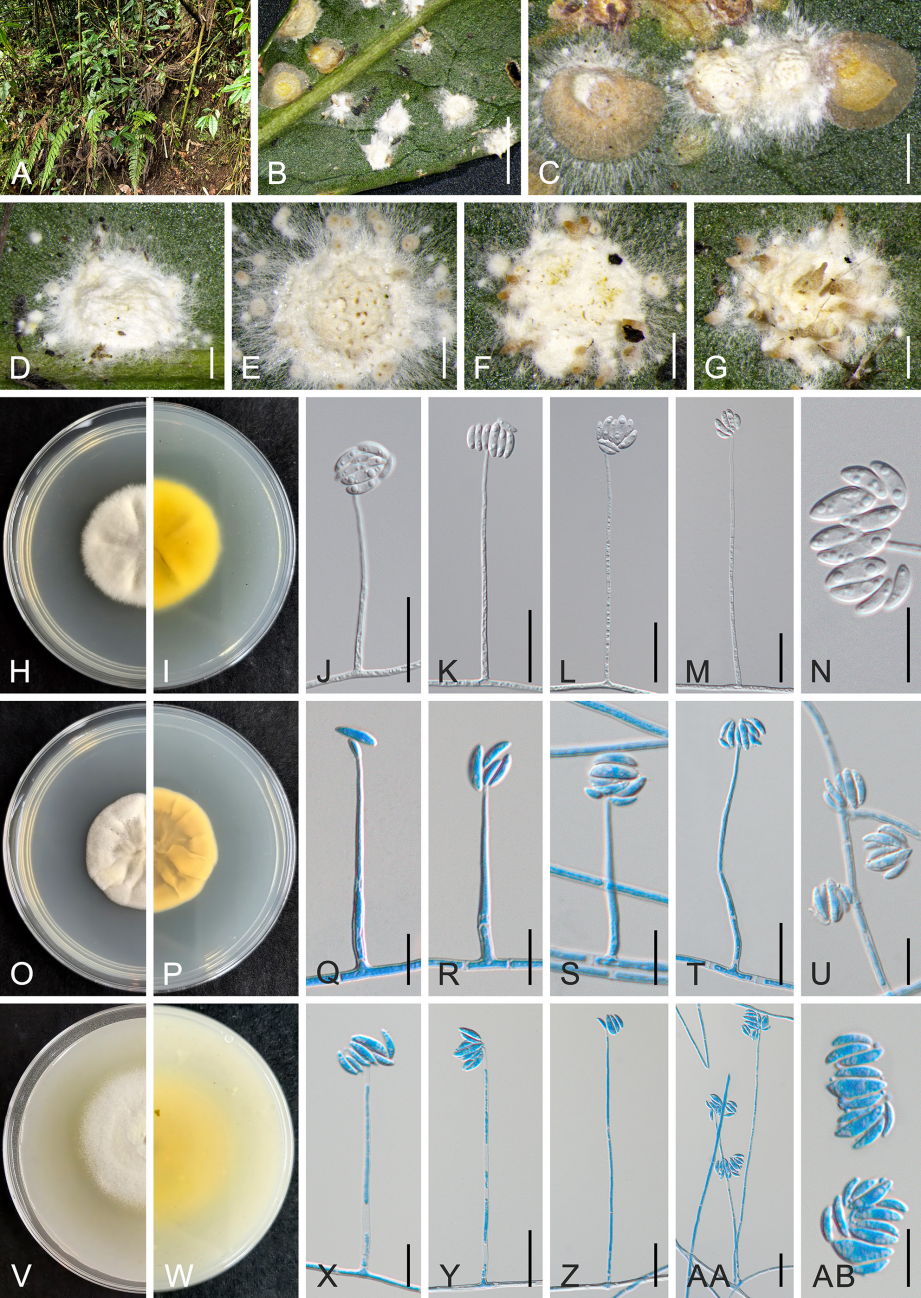
*Conoideocrella tenuis* (SICAU 25-0209, living culture SICAUCC 25-0177). **A** Habitat; **B–G** Telemorphic stromata containing perithecia; **H, I** Colonies on PDA after 21 d; **J–N** Conidiophores bearing phialides and conidia on PDA; **O, P** Colonies on SDAY/4 after 21 d; **Q–U** Conidiophores bearing phialides and conidia on SDAY/4; **V, W** Colonies on OA after 21 d; **X–AB** Conidiophores bearing phialides and conidia on OA. Scale bars: 1000 µm (**B**); 200 µm (**C–G**); 20 µm (**J–M, Y–AA**); 10 µm (**N, Q–U, AB**).

Colonies on SDAY/4 reaching 24–27 mm diam after 21 d at 25 °C, milky white to greyish white, circular, flat, cottony to felty with moderate aerial mycelium, closely appressed to the agar surface, intensely radially folded, margin entire, reverse pale yellowish brown. ***Conidiophores*** solitary, erect, straight or slightly curved, arising directly from aerial and substratal mycelium, usually reduced to single phialides, unbranched, with 1–3 transverse septa, gradually tapering from the base to the apex, hyaline, smooth-walled. ***Phialides*** cylindrical or subulate, lateral, (45–)70–100(–120) × 1.8–2.6 µm. ***Conidia*** fusiform and slightly curved, arranged in a group of 3–6 at the neck apex, aseptate, hyaline, smooth-walled, with guttules, (6–)7–9.5(–10.3) × 1.8–3 µm. ***Chlamydospores*** not observed.

Colonies on OA reaching 30–33 mm diam after 21 d at 25 °C, creamy white, circular, flat, floccose, aerial mycelium sparse, arranged in radial lines, closely appressed to the agar surface, margin filiform, reverse pale yellow. ***Conidiophores*** solitary, erect, straight, arising directly from aerial mycelium, usually reduced to single phialides, unbranched, with 1–4 transverse septa, gradually tapering from the base to the apex, hyaline, smooth-walled, with cell walls usually thinner than those of vegetative hyphae. ***Phialides*** cylindrical or subulate, lateral, (100–)113–167(–172) × 1.8–2.4 µm. ***Conidia*** fusiform and slightly curved, arranged in a group of 4–10 at the neck apex, aseptate, hyaline, smooth-walled, with guttules, 6–9.5(–10.7) × 1.7–3(–3.6) µm. ***Chlamydospores*** not observed.

##### Hosts.

Whiteflies (*Aleyrodidae*, *Hemiptera*), scale insects (*Coccidae*, *Hemiptera*).

##### Known distribution.

Belize, China (Yunnan, Sichuan), Japan, Sri Lanka, and Thailand.

##### Material examined.

CHINA • Sichuan Province, Meishan City, Hongya County, Gaomiao Town, Huayuan Village, 29°35'34"N, 103°14'07"E, on scale insects on the abaxial surface of living leaves of *Camellia sinensis*, 18 Mar. 2025, Feng Liu, SICAU 25-0209, living culture SICAUCC 25-0177; • ibid. SICAU 25-0211, living culture SICAUCC 25-0179; Ya’an City, Yingjing County, Niubei Mountain Town, Nanmu Village, 29°37'24"N, 103°11'57"E, on scale insects on the abaxial surface of living leaves of *Camellia sinensis*, 19 Jul. 2025, Feng Liu, SICAU 25-0210, living culture SICAUCC 25-0178.

##### Notes.

In our three-gene phylogenetic analysis, new strains (SICAUCC 25-0177 to 25-0179) grouped with *Conoideocrella tenuis* (NHJ 6791, NHJ 6293, and NHJ 345.01), forming a robust clade with strong statistical support (MLBS = 100%, BPP = 1.00; Fig. 1). *Conoideocrella tenuis* was initially found on scale insects (*Hemiptera*) on the underside of living leaves of dicotyledonous plants in pine forests in Thailand (Hywel-Jones 1993), and Wang et al. (2024b) subsequently reported this species in Yunnan Province, China. The collection SICAU 25-0210 and strain SICAUCC 25-0177 exhibit morphological characteristics similar to those described by Wang et al. (2024b), demonstrating comparable dimensions in perithecia (495–600 × 170–235 µm vs 190–500 × 160–270 µm), asci (275–370 × 4–6.6 µm vs 190–480 × 3.3–5.5 µm), phialides (80–122 × 1.7–2.4 µm vs 16.3–149.4 × 0.6–2.4 µm), and conidia (7–9.5 × 1.8–3 µm vs 6.1–12.5 × 1.3–2.3 µm). Consequently, based on the phylogenetic and morphological similarities, we identified our specimens as *C. tenuis*, representing a new geographical record for this species in Sichuan Province, China.

#### Conoideocrella violaceomarginata

Taxon classificationAnimaliaHypocrealesClavicipitaceae

Feng Liu & Chun L. Yang sp. nov.

0DB66BAF-F0BA-5AC5-B212-2976F6BE01BD

Index Fungorum: IF902138

Fig. 8

##### Etymology.

In reference to the distinct violet-pigmented ring at the stromatal margin.

##### Diagnosis.

Similar to *Conoideocrella fenshuilingensis* in having comparable morphology of stromata, conidiophores, and conidia, but *C. violaceomarginata* differs by its smaller stromata with different color, shorter phialides, larger conidia, and significant nucleotide differences in *rpb*1 and *tef*1-α.

##### Type.

CHINA • Sichuan Province, Ya’an City, Tianquan County, Xiaohe Town, 30°06'34"N, 102°44'17"E, on scale insects on the abaxial surface of living leaves of *Camellia sinensis* (L.) Kuntze, 25 Sep. 2025, Feng Liu, **holotype**SICAU 25-0213, culture ex-type SICAUCC 25-0181.

##### Description.

Parasitic on the scale insects (*Coccidae*, *Hemiptera*) from *Camellia sinensis* (L.) Kuntze (*Theaceae*). ***Teleomorph***: Not observed. ***Anamorph***: hirsutella-like. ***Stromata*** pale brown to yellowish-brown when young, turning yellowish-orange in the center and bearing a conspicuous violaceous pigmented ring at the margin at maturity, subcircular to pulvinate, scattered, cottony, mycelium arranged in radial lines, completely covering the insect hosts, 0.2–0.4 mm in diameter, intense sporulation with powdery in the center.

**Figure 8.**
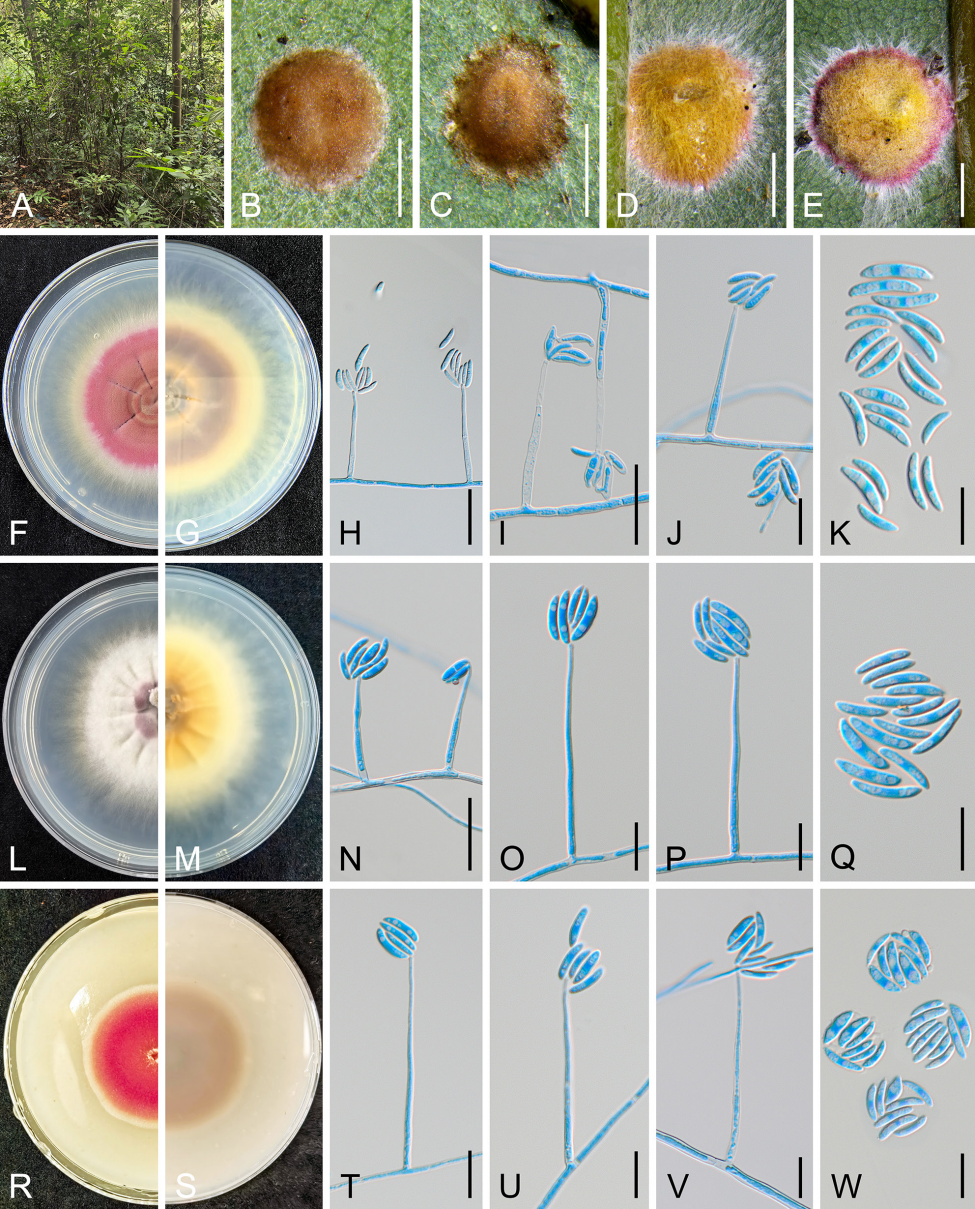
*Conoideocrella violaceomarginata* (holotype SICAU 25-0213, living culture SICAUCC 25-0181). **A** Habitat; **B–E** Anamorphic stroma containing conidiomata on the host; **F, G** Colonies on PDA after 21 d; **H–K** Conidiophores bearing phialides and conidia on PDA; **L, M** Colonies on SDAY/4 after 21 d; **N–Q** Conidiophores bearing phialides and conidia on SDAY/4; **R, S** Colonies on OA after 21 d; **T–W** Conidiophores bearing phialides and conidia on OA. Scale bars: 1000 µm (**B–E**); 20 µm (**H, I, N**); 10 µm (**J, K, O–Q, T–W**).

##### Culture characteristics.

Colonies on PDA reaching 42–45 mm diam after 21 d at 25 °C, dull pinkish brown in the center, powdery while sporulating, creamy white at periphery, circular, flat, floccose to felty, abundant aerial mycelium, generating several concentric rings in the center, radially folded, margin entire, reverse grayish brown. ***Conidiophores*** solitary, erect, straight, arising directly from aerial mycelium, usually reduced to single phialides, unbranched, 1–2-septate, gradually tapering towards the apex, hyaline, smooth-walled. ***Phialides*** narrowly cylindrical or subulate, lateral, (30–)32–42(–45) × 2–2.9 µm. ***Conidia*** fusiform and slightly curved, arranged in slimy heads, 0–1-septate, hyaline, smooth-walled, (7.6–)8–11.5(–12.8) × 1.8–2.6 µm. ***Chlamydospores*** not observed.

Colonies on SDAY/4 reaching 40–43 mm diam after 21 d at 25 °C, vinaceous buff in the center, powdery while sporulating, creamy white at periphery, circular, flat, floccose to cottony, abundant aerial mycelium, radially folded, margin entire, reverse pale yellow. ***Conidiophores*** solitary, erect, straight, arising directly from aerial mycelium, usually reduced to single phialides, unbranched, 1–2-septate, gradually tapering towards the apex, hyaline, smooth-walled. ***Phialides*** narrowly cylindrical or subulate, lateral, (32–)37–42(–46) × 2–3 µm. ***Conidia*** fusiform and slightly curved, arranged in slimy heads, 0–1-septate, hyaline, smooth-walled, (7.5–)8.5–12 × 1.9–2.8 µm. ***Chlamydospores*** not observed.

Colonies on OA reaching 20–30 mm diam after 21 d at 25 °C, bright red, circular, creamy white at periphery, flat, membranous with sparse aerial mycelium, arranged in radially lines, margin entire, reverse pale fawn. ***Conidiophores*** solitary, erect, straight, arising directly from aerial and substratal mycelium, usually reduced to single phialides, unbranched, 1–2-septate, gradually tapering towards the apex, hyaline, smooth-walled. ***Phialides*** narrowly cylindrical or subulate, lateral, 40–45 × 1.9–2.5 µm. ***Conidia*** fusiform and slightly curved, arranged in slimy heads, 0–1-septate, hyaline, smooth-walled, (7.5–)9–12(–14.7) × 1.8–2.8 µm. ***Chlamydospores*** not observed.

##### Hosts.

Scale insects (*Coccidae*, *Hemiptera*).

##### Known distribution.

China (Sichuan).

##### Additional specimen examined.

CHINA • Sichuan Province, Ya’an City, Tianquan County, Xiaohe Town, 30°06'34"N, 102°44'17"E, on scale insects on the abaxial surface of living leaves of *Camellia sinensis* (L.) Kuntze, 25 Sep. 2025, Feng Liu, SICAU 25-0214, living culture SICAUCC 25-0182.

##### Notes.

In the phylogenetic analyses of this study, *Conoideocrella violaceomarginata* formed a sister clade to *C. fenshuilingensis* (YHH CFFSL2310002 and YHH CFFSL2310003) with 99% MLBS/1.00 BPP statistical support (Fig. 1). Morphologically, *C. violaceomarginata* (SICAU 25-0213) is distinct from the holotype of *C. fenshuilingensis* (YHH CFFSL2310002) in having smaller stromata (0.2–0.4 mm vs. 3.0–3.4 mm), and different color (yellowish-orange vs. snow-white) (Wang et al. 2024b). In this study, compared to *C. fenshuilingensis* (SICAUCC 25-0180), *C. violaceomarginata* (SICAUCC 25-0181) is distinguished by its shorter phialides (32–42 × 2–2.9 µm vs. 40–67 × 1.3–2.1 µm), and larger conidia (8–11.5 × 1.8–2.6 µm vs. 5.8–8.5 × 1.7–2.4 µm) on PDA. Moreover, the nucleotide differences between *C. violaceomarginata* (SICAUCC 25-0181) and *C. fenshuilingensis* (YHH CFFSL2310002) are 6.57% (47/715, 0 gaps) in *rpb*1, and 4.82% (44/913, 0 gaps) in *tef*1-α. Consequently, based on combined morphological and molecular evidence, we propose *C. violaceomarginata* as a new species.

#### Hypocrella cinnamomum

Taxon classificationAnimaliaHypocrealesClavicipitaceae

Feng Liu & Chun L. Yang sp. nov.

5368C704-B368-51BA-A333-76AC99CA7446

Index Fungorum: IF903581

Fig. 9

##### Etymology.

In reference to the generic name of host plants.

##### Diagnosis.

Similar to *Hypocrella limushanensis* and *H. calendulina* in having comparable morphology of conidiomata, conidiophores, and conidia, but *H. cinnamomum* differs by its larger conidiomata, shorter phialides, and smaller conidia.

##### Type.

CHINA • Sichuan Province, Ya’an City, Baoxing County, Longdong Town, Laocang New Village, 30°28'50"N, 102°42'57"E, on scale insects on the adaxial surface of living leaves of *Cinnamomum japonicum* Sieb., 23 Sep. 2025, Feng Liu, **holotype**SICAU 25-0224, culture ex-type SICAUCC 25-0192.

##### Description.

Parasitic on scale insects (*Coccidae*, *Hemiptera*) from *Cinnamomum japonicum* Sieb. (*Lauraceae*). ***Teleomorph***: Not observed. ***Anamorph*: *Stromata*** pulvinate to discoid, discoid to thickened pulvinate, pale yellow when young, turning reddish-brown to dark brown at maturity, superficial, 1–2.5 mm in diameter. ***Conidiomata*** pycnidial, subglobose, pale yellow to orange red, scattered to clustered, circularly arranged towards the margin of stroma, slightly concave, 12–20 conidiomata per stroma, 440–670 × 350–520 µm, conidiogenous layer composed of a thick, compact palisade of phialides. ***Ostioles*** sunken, orange yellow to orange red. ***Conidiomata walls*** 75–105 µm wide, unequal thickness, thicker in top regions, multi-layered, outer stratum 35–60 µm wide, pale yellow, composed of loosely interwoven prosenchymatous cells, forming a spongy texture; inner stratum 45–65 µm wide, gradually turning hyaline toward the lumen, composed of pseudoparenchymatous cells, arranged in *textura angularis* to *textura prismatica*, indistinguishable from conidiogenous cells. ***Paraphyses*** linear, filiform, curved, septate, hyaline, (97–)110–140(–165) × 1.4–2.1 µm. ***Phialides*** cylindrical, slightly tapering towards the tip, hyaline, smooth-walled, 9.3–15(–26) × 1.6–2.6 µm. ***Conidia*** fusoid with acute ends, slightly curved, unicellular, hyaline, smooth-walled, with small guttules, 7.8–10 × 2–2.6 µm.

**Figure 9.**
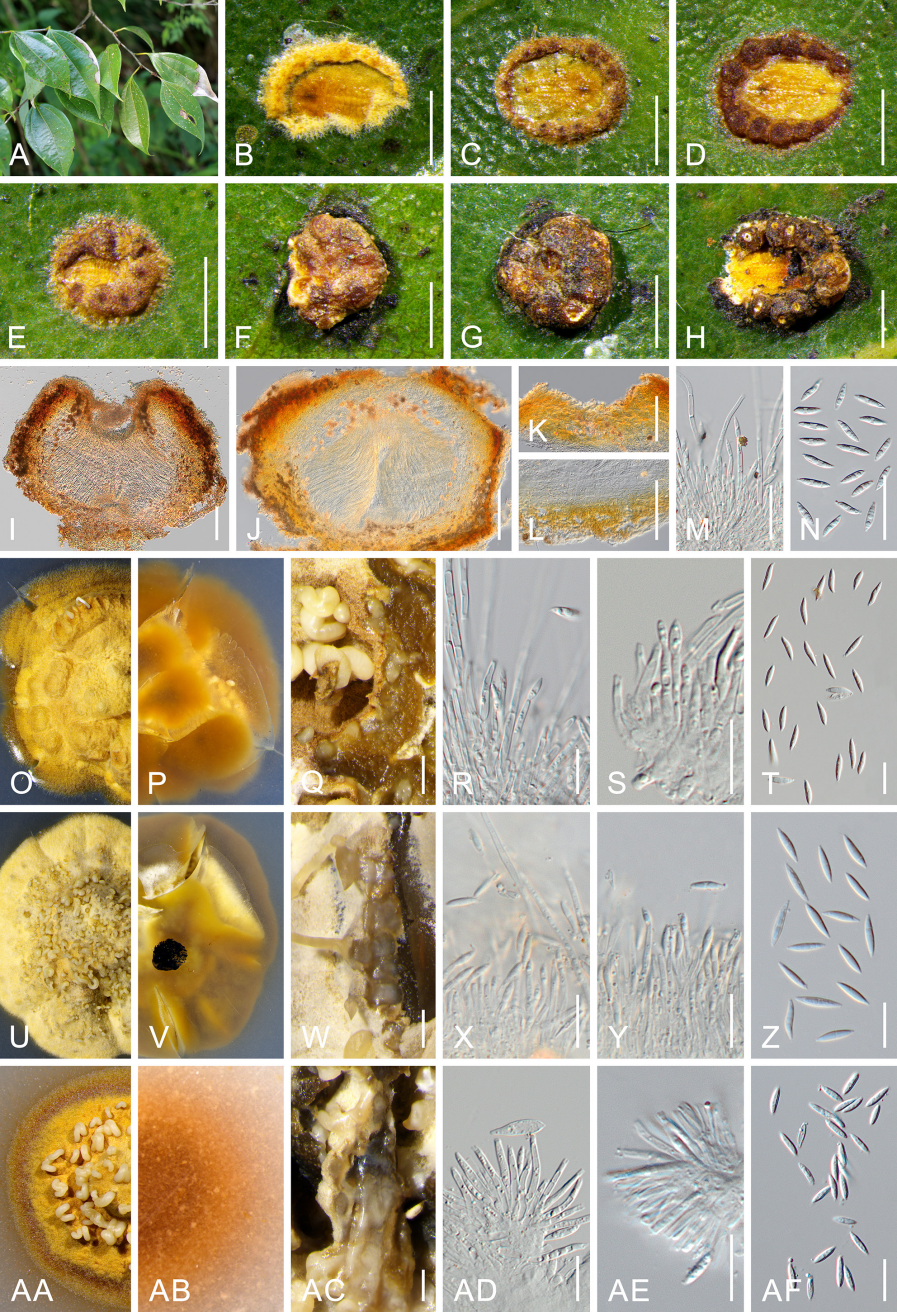
*Hypocrella cinnamomum* (holotype SICAU 25-0224, culture ex-type SICAUCC 25-0192). **A** Habitat; **B–H** Anamorphic stroma containing conidiomata on the host; **I, J** Vertical section through conidiomata; **K** Section of the ostiole part; **L** Section through conidiomatal wall; **M** Conidiogenous layer with conidiogenous cells and paraphyses; **N** Conidia; **O–Q** Colonies on PDA after 50 d, showing slimy conidial masses and cirrhi oozing from pycnidial-acervular conidiomata; **R–T** Conidiophores bearing phialides and conidia on PDA; **U–W** Colonies on SDAY/4 after 50 d, showing slimy conidial masses and cirrhi oozing from pycnidial-acervular conidiomata; **X–Z** Conidiophores bearing phialides and conidia on SDAY/4; **AA–AC** Colonies on OA after 50 d, showing slimy conidial masses and cirrhi oozing from pycnidial-acervular conidiomata; **AD–AF** Conidiophores bearing phialides and conidia on OA. Scale bars: 1000 µm (**B–H**); 500 µm (**Q, W, AC**); 100 µm (**I–L**); 20 µm (**M, N**); 10 µm (**R–T, X–Z, AD–AF**).

##### Culture characteristics.

Colonies on PDA reaching 8–11 mm diam after 50 d at 25 °C, yellowish-orange to golden yellow, circular, slightly raised, felty, with moderately aerial mycelium, closely appressed to the agar surface, radially folded, margin dentate, reverse russet brown. Conidial masses abundant, cream white. ***Paraphyses*** linear, filiform, flexuous, septate, hyaline, 100–150 × 1.4–2.2 µm. ***Phialides*** cylindrical, slightly tapering towards the tip, hyaline, smooth-walled, (9.2–)12–15(–20.2) × 1.5–2.1 µm. ***Conidia*** fusoid with acute ends, slightly curved, unicellular, hyaline, smooth-walled, 7.5–10 × 1.8–2.3 µm.

Colonies on SDAY/4 reaching 10–13 mm diam after 50 d at 25 °C, cream white to pale yellow, circular, slightly raised, felty, with moderately aerial mycelium, closely appressed to the agar surface, slightly radially folded, margin crenate, reverse pale yellowish-brown. Conidial masses abundant, cream white to pale yellow. ***Paraphyses*** linear, filiform, flexuous, septate, hyaline, 100–160 × 1.4–2.1 µm. ***Phialides*** cylindrical, slightly tapering towards the tip, hyaline, smooth-walled, 10–15.6 × 1.2–1.6 µm. ***Conidia*** fusoid to narrowly fusiform, with acute ends, sometimes slightly curved, unicellular, hyaline, smooth-walled, 7.3–11 × 1.7–2.4 µm.

Colonies on OA reaching 8–10 mm diam after 50 d at 25 °C, bright yellow in the center, red-brown at the margin, circular, flat, felty, with moderate aerial mycelium, arranged in radially lines, closely appressed to the agar surface, margin entire, reverse deep russet red-brown. Conidial masses abundant, cream white. ***Paraphyses*** linear, filiform, flexuous, septate, hyaline, 93–145 × 1.2–2 µm. ***Phialides*** cylindrical, slightly tapering towards the tip, hyaline, smooth-walled, 10–17 × 1.2–1.5 µm. ***Conidia*** fusoid with acute ends, sometimes slightly curved, unicellular, hyaline, smooth-walled, 6–10.6 × 1.5–1.9 µm.

##### Hosts.

Scale insects (*Coccidae*, *Hemiptera*).

##### Known distribution.

China (Sichuan).

##### Additional specimen examined.

CHINA • Sichuan Province, Ya’an City, Baoxing County, Longdong Town, Laocang New Village, 30°28'50"N, 102°42'57"E, on scale insects on the adaxial surface of living leaves of *Cinnamomum japonicum* Sieb., 23 Sep. 2025, Feng Liu, SICAU 25-0225, living culture SICAUCC 25-0193.

##### Notes.

In our phylogenetic analysis, our strains (SICAUCC 25-0192 and SICAUCC 25-0193) formed a sister lineage with *H. cf. discoidea* (I93-901D and I95-901D), *H. limushanensis* (YHH 2303015 and YHH 2303016), and *H. calendulina* (BCC 20309) with 85% MLBS and 0.97 BPP statistical support (Fig. 1). *H. cinnamomum* can be distinguished from *H. limushanensis* by its subglobose, pale yellow to orange-red, larger conidiomata (440–670 × 350–520 µm vs. 190–370 × 140–320 µm) (Wang et al. 2025b). In contrast, the conidiomata, paraphyses, and conidia of *H. cinnamomum* are larger (440–670 × 350–520 µm vs. 200–390 × 300–400 µm), shorter (9.3–15 × 1.6–2.6 µm vs. up to 65 × 1.5 µm), and smaller (7.8–10 × 2–2.6 µm vs. 12.5–15 × 2.5–3.8 µm) than those of *H. calendulina* (Mongkolsamrit et al. 2009). However, comprehensive morphological comparisons between *H. cinnamomum* and *H. cf. discoidea* were not feasible due to the absence of documented morphological data for *H. cf. discoidea*. Thus, based on combined phylogenetic and morphological evidence, *H. cinnamomum* is hereby proposed as a new species.

#### Helicocollum bambusae

Taxon classificationAnimaliaHypocrealesClavicipitaceae

Feng Liu & Chun L. Yang sp. nov.

CC37C959-7DD2-5FC7-81CA-D9FC6745FBA4

Index Fungorum: IF903582

Fig. 10

##### Etymology.

In reference to the generic name of host plants.

##### Diagnosis.

Similar to *Helicocollum krabiensis* in having comparable morphology of stromata, conidiophores, and conidia, but *Helicocollum bambusae* differs by its slimmer phialides, longer helical necks, and significant nucleotide differences in LSU, *tef*1-α, and *rpb*1.

##### Type.

CHINA • Sichuan Province, Yibin City, Pingshan County, Longshang Road, 28°50'31"N, 104°01'28"E, on scale insects on the abaxial surface of living leaves of *Bambusa emeiensis* L. C. Chia & H. L. Fung, 19 Mar. 2025, Feng Liu, **holotype**SICAU 25-0222, culture ex-type SICAUCC 25-0190.

##### Description.

Parasitic on scale insects (*Coccidae*, *Hemiptera*) from *Bambusa emeiensis*. ***Teleomorph***: Not observed. ***Anamorph*: *Stromata*** pulvinate or tomentose, central slightly raised, superficial, subcircular, scattered to clustered, pale yellowish to orange yellow, mycelium arranged in radial lines, completely covering the host, 0.5–2 mm in diameter, intense sporulation with granular conidial masses in the center.

**Figure 10.**
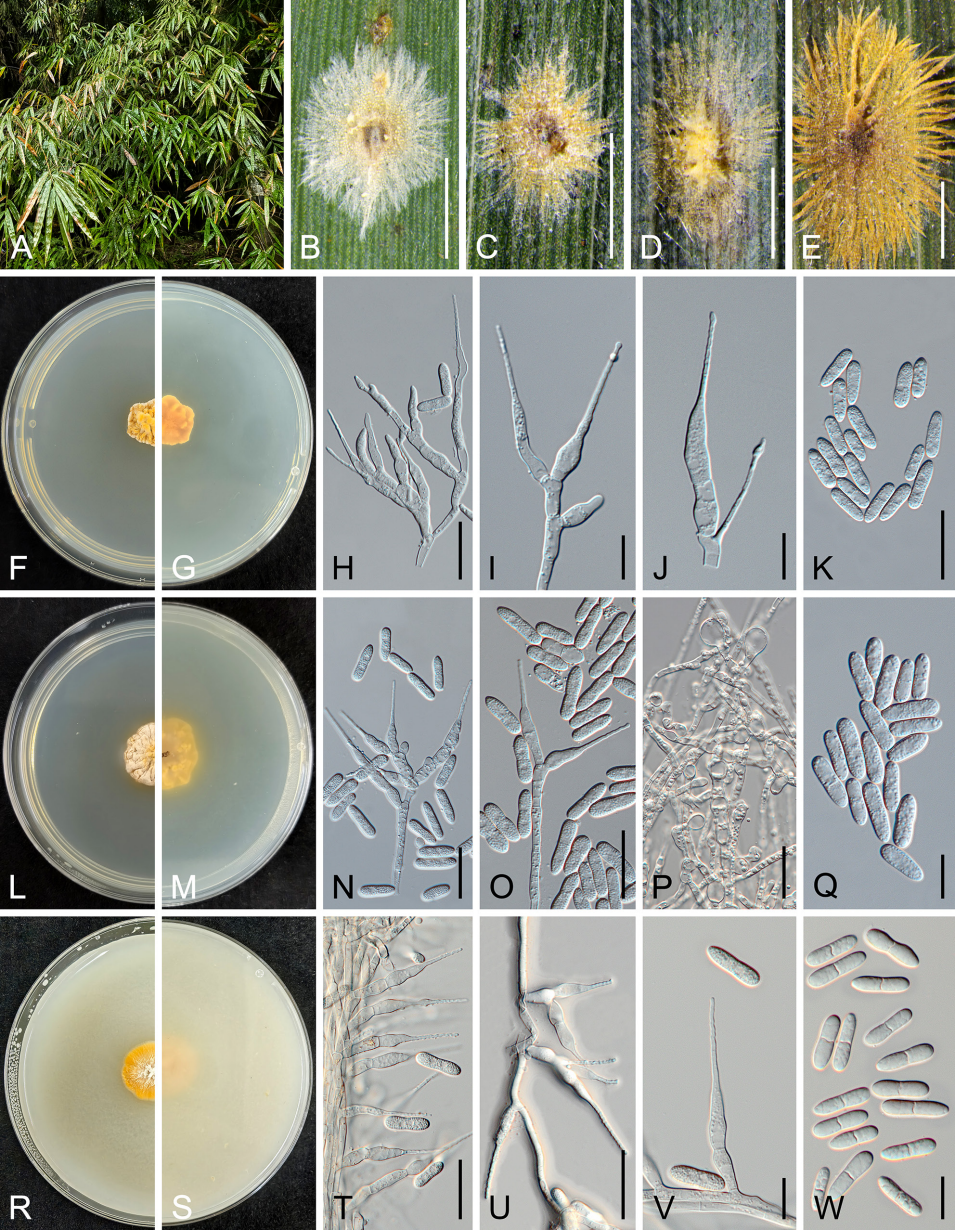
*Helicocollum bambusae* (holotype SICAU 25-0222, culture ex-type SICAUCC 25-0190). **A** Habitat; **B–E** Anamorphic stroma containing conidiomata on the host; **F, G** Colonies on PDA after 30 d; **H–K** Conidiophores bearing phialides and conidia on PDA; **L, M** Colonies on SDAY/4 after 30 d; **N–Q** Conidiophores bearing phialides, conidia, and chlamydospores on SDAY/4; **R, S** Colonies on OA after 30 d; **T–W** Conidiophores bearing phialides and conidia on OA. Scale bars: 2000 µm (**B**); 500 µm (**C**); 300 µm (**D, E**); 20 µm (**H, K, N–P, T, U**); 10 µm (**I, J, Q, V, W**).

##### Culture characteristics.

Colonies on PDA reaching 8–10 mm diam after 30 d at 25 °C, pale yellow to yellow brown, raised, felty to pulvinate with sparse aerial mycelium, radially folded, margin crenate, reverse yellow brown. ***Conidiophores*** solitary, erect, straight or slightly curved, arising directly from the substratal mycelium, usually reduced to single phialides, verticillate, unbranched or basitonously branched, with 2–5 septa at middle and base, hyaline, smooth-walled. ***Phialides*** mono- or polyphialidic, lateral or terminal, orthotropic, flask-shaped or pear-shaped, straight or slightly curved, hyaline, thick-, smooth-walled, entire phialides 30–42(–48) × 3–5.6 µm, slightly helical necks present, 15–23.5(–26) × 1–1.6 µm. ***Conidia*** cylindrical, 1-septate, slightly constricted at septum, hyaline, smooth-walled, with many guttules, (10.5–)12–15.5 × 3.3–4.6 µm. ***Chlamydospores*** not observed.

Colonies on SDAY/4 reaching 10–12 mm diam after 30 d at 25 °C, grayish white to grayish yellow, raised, felty to pulvinate with sparse aerial mycelium, radially folded, margin crenate, reverse grayish yellow, with slightly pale-yellow pigment. ***Conidiophores*** solitary, erect, straight, arising directly from the substratal mycelium, usually reduced to single phialides, verticillately, unbranched or basitonously branched, with 2–3 septa at middle and base, hyaline, smooth-walled. ***Phialides*** mono- or polyphialidic, lateral or terminal, orthotropic, flask-shaped or pear-shaped, straight or slightly curved, hyaline, thick-, smooth-walled, entire phialides 26–40(–43) × 3.3–5 µm, with slightly helical necks, 12.5–20(–21.6) × 1–1.7 µm. ***Conidia*** cylindrical, 1-septate, slightly constricted at septum, hyaline, smooth-walled, with many guttules, 12.5–16 × 3.6–5 µm. ***Chlamydospores*** present, obovate to globose, terminal, or intercalary on hyphae, or lateral on short stipes, hyaline, smooth-, thick-walled.

Colonies on OA reaching 7–10 mm diam after 30 d at 25 °C, cream white to orange yellow, circular, flat, membranous to floccose with moderate aerial mycelium, arranged in radially lines, closely appressed to the agar surface, margin crenate, reverse pale orange yellow. ***Conidiophores*** solitary, erect, straight or irregularly curved, arising directly from aerial and substratal mycelium, or from ropes formed by the mycelium, usually reduced to single phialides, unbranched or basitonously branched, with 2–3 septa at middle and base, hyaline, smooth-walled. ***Phialides*** mono- or polyphialidic, lateral or terminal, orthotropic, flask-shaped or pear-shaped, straight or slightly curved, hyaline, thick-, smooth-walled, entire phialides (24–)27–45(–48) × 3.2–5 µm, slightly helical necks present, 15–20(–21.5) × 1–1.6 µm. ***Conidia*** cylindrical, 1-septate, slightly constricted at septum, hyaline, smooth-walled, with many guttules, 13.3–17 × 3.2–5.2 µm. ***Chlamydospores*** not observed.

##### Hosts.

Scale insects (*Coccidae*, *Hemiptera*).

##### Known distribution.

China (Sichuan).

##### Additional specimen examined.

CHINA • Sichuan Province, Yibin City, Pingshan County, Longshang Road, 28°50'31"N, 104°01'28"E, on scale insects on the abaxial surface of living leaves of *Bambusa emeiensis* L. C. Chia & H. L. Fung, 19 Mar. 2025, Feng Liu, SICAU 25-0223, living culture SICAUCC 25-0191.

##### Notes.

In the phylogenetic analyses of this study, *Helicocollum bambusae* (SICAUCC 25-0190 and SICAUCC 25-0191) formed a sister clade with *He. krabiensis* (BCC 68582, BCC 68583, and BCC 68584) with 100% MLBS/1.00 BPP statistical support (Fig. 1). Morphologically, *He. bambusae* can be distinguished from *He. krabiensis* by its slimmer entire phialides (27–45 × 3.2–5 µm vs. 16.5–28 × 4.5–7.5 µm), and longer helical necks (15–20 µm vs. 2.5–5.5 µm) on PDA (Luangsa-ard et al. 2017). Nucleotide comparison between *He. bambusae* (SICAUCC 25-0190) and the ex-type strain of *He. krabiensis* (BCC 68582) reveals 1.3% (10/769, 10 gaps), 6.7% (59/881, 0 gaps), and 6.23% (39/626, 0 gaps) differences in LSU, *tef*1-α and *rpb*1, respectively. Consequently, based on combined morphological and phylogenetic evidence, we propose *He. bambusae* as a new species.

#### Regiocrella sichuanensis

Taxon classificationAnimaliaHypocrealesClavicipitaceae

Feng Liu & Chun L. Yang sp. nov.

1DF09138-738B-5990-AAC8-28892B4443BB

Index Fungorum: IF903583

Fig. 11

##### Etymology.

Name refers to Sichuan Province where it was collected.

##### Diagnosis.

Similar to *Regiocrella camerunensis* in having comparable morphology of stromata, conidiophores, and conidia, but *R. sichuanensis* differs by its shape of stromata, narrower phialides, smaller conidia, and significant nucleotide differences in LSU, *rpb*1 and *tef*1-α.

##### Type.

CHINA • Sichuan Province, Ya’an City, Yingjing County, Longcanggou Town, Kuaile Village, 29°40'38"N, 102°51'51"E, on scale insects on the abaxial surface of living leaves of *Camellia sinensis* (L.) Kuntze, 18 Jul. 2025, Feng Liu, **holotype**SICAU 25-0220, culture ex-type SICAUCC 25-0188.

##### Description.

Parasitic on scale insects (*Coccidae*, *Hemiptera*) from *Camellia sinensis* (L.) Kuntze (*Theaceae*). ***Teleomorph***: Not observed. ***Anamorph*: *Stromata*** thickened pulvinate to hemi-globose, superficial, subcircular, scattered, dark brown to blackish when young, turning orange-yellow at intense sporulation, thin, grayish white mycelia completely covering the host, powdery while sporulating around the stroma. ***Conidia*** ellipsoid or cylindrical, rounded at both ends, aseptate, hyaline, smooth-walled, 3.3–4.3 × 1.6–2 µm.

**Figure 11.**
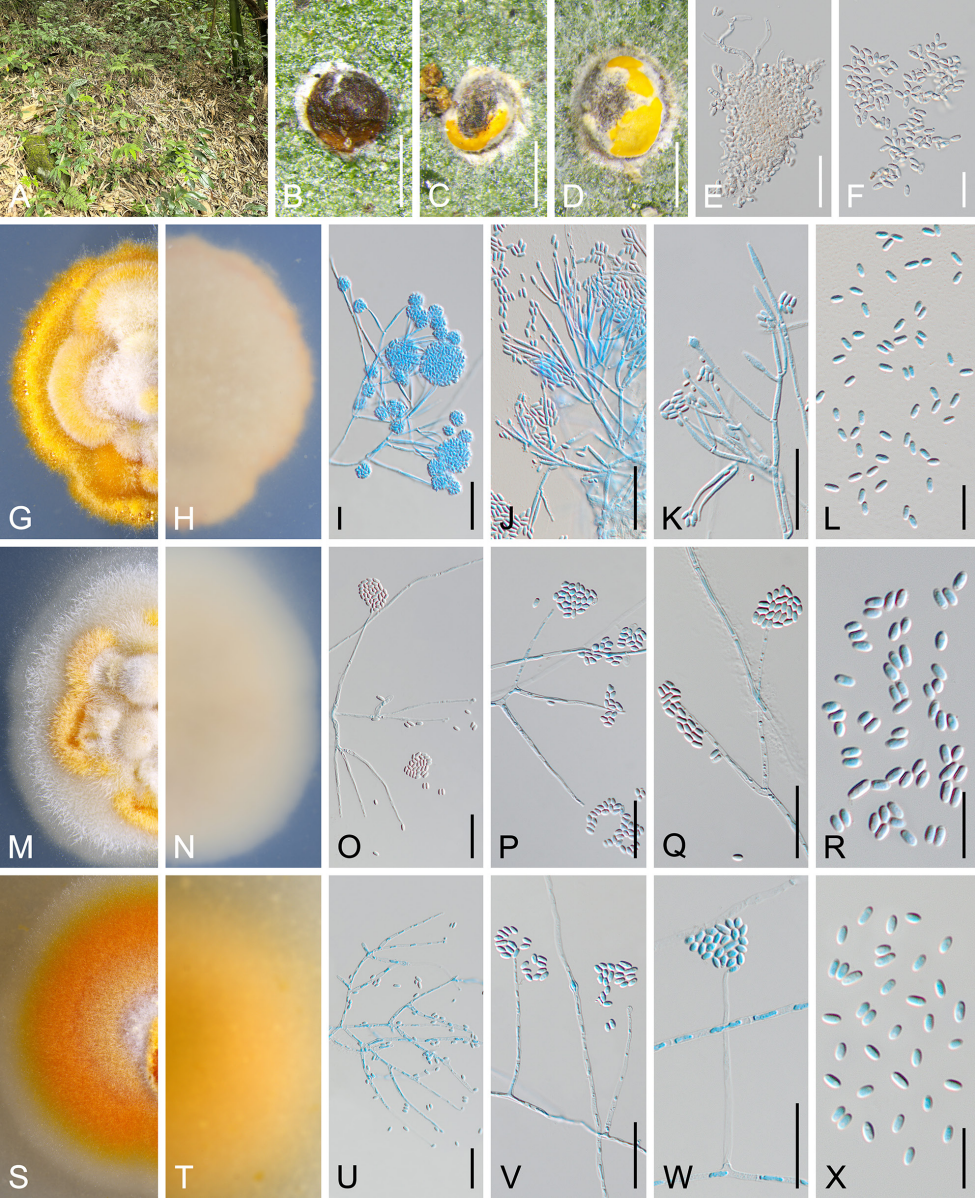
*Regiocrella sichuanensis* (holotype SICAU 25-0220, culture ex-type SICAUCC 25-0188). **A** Habitat; **B–D** Anamorphic stroma containing conidiomata on the host; **E, F** Conidia on insect host; **G, H** Colonies on PDA after 30 d; **I–L** Conidiophores bearing phialides and conidia on PDA; **M, N** Colonies on SDAY/4 after 30 d; **O–R** Conidiophores bearing phialides and conidia on SDAY/4; **S, T** Colonies on OA after 30 d; **U–X** Conidiophores bearing phialides and conidia on OA. Scale bars: 1000 µm (**B–D**); 20 µm (**E, F, I–K, O–Q, U–W**); 10 µm (**L, R, X**).

##### Culture characteristics.

Colonies on PDA reaching 7–10 mm diam after 30 d at 25 °C, creamy white in the center, bright yellow at periphery, slightly irregular, flat to slightly raised, fluffy with abundant aerial mycelium forming concentric rings, slightly folded, margin crenate, reverse pale beige. ***Conidiophores*** aggregated, erect, straight, arising directly from aerial mycelium, mostly repeatedly verticillately branched, bearing up to 1–3 whorls with 1–3 phialides per node, with a single basal septum, hyaline, smooth-walled. ***Phialides*** cylindrical or subulate, lateral or terminal, gradually tapering at top, 9–14(–16) × 1.2–1.7 µm, arranged in slimy heads. ***Conidia*** ellipsoid or cylindrical, rounded at both ends, aseptate, hyaline, smooth-walled, 2.8–3.7 × 1.4–1.7 µm. ***Chlamydospores*** not observed.

Colonies on SDAY/4 reaching 12–15 mm diam after 30 d at 25 °C, creamy white to pale yellow in the center, creamy white at periphery, circular, flat to slightly raised, fluffy with abundant aerial mycelium forming irregular concentric rings, folded, margin crenate, reverse pale creamy. ***Conidiophores*** solitary, erect, straight, arising directly from aerial mycelium, basitonously branched or unbranched and reduced to single phialides, with a single basal septum, hyaline, smooth-walled. ***Phialides*** cylindrical or subulate, lateral or terminal, gradually tapering at top, (22–)30–43(–48) × 1.5–2.3 µm, arranged in slimy heads. ***Conidia*** ellipsoid or cylindrical, rounded at both ends, aseptate, hyaline, smooth-walled, 2.2–3.6 × 1.5–2 µm. ***Chlamydospores*** not observed.

Colonies on OA reaching 12–15 mm diam after 30 d at 25 °C, orange yellow in the center, yellowish orange at periphery, circular, flat, membranous with sparse aerial mycelium, arranged in radially lines, closely appressed to the agar surface, margin filiform, reverse pale orange yellow, faint pigment diffusing locally. ***Conidiophores*** solitary, erect, straight, arising directly from the agar surface and substratal mycelium, unbranched, usually reduced to single phialides, or repeatedly verticillately branched, bearing 1–3 whorls with 1–3 phialides per node, with a single basal septum, gradually tapering at top, hyaline, smooth-walled. ***Phialides*** cylindrical or subulate, lateral or terminal, gradually tapering at top, (26.5–)30–42(–46) × 1.4–2.2 µm, arranged in slimy heads. ***Conidia*** ellipsoid or cylindrical, rounded at both ends, aseptate, hyaline, smooth-walled, 2.7–3.7 × 1.5–1.9 µm. ***Chlamydospores*** not observed.

##### Hosts.

Scale insects (*Coccidae*, *Hemiptera*).

##### Known distribution.

China (Sichuan).

##### Additional specimen examined.

CHINA • Sichuan Province, Ya’an City, Yingjing County, Longcanggou Town, Kuaile Village, 29°40'38"N, 102°51'51"E, on scale insects on the abaxial surface of living leaves of *Camellia sinensis* (L.) Kuntze, 18 Jul. 2025, Feng Liu, SICAU 25-0221, living culture SICAUCC 25-0189.

##### Notes.

In the phylogenetic analyses of this study, *Regiocrella sichuanensis* is most closely related to *R. camerunensis* with high statistical support (100% MLBS/1.00 BPP; Fig. 1). *Regiocrella sichuanensis* is morphologically distinguishable from *R. camerunensis* by its pulvinate to hemiglobose stromata, narrower phialides (9–14 × 1.2–1.7 µm vs. 10.2–14.0 × 2.0–2.5 µm), and smaller conidia (2.8–3.7 × 1.4–1.7 µm vs. 4.0–4.5 × 2.0–2.2 µm) on PDA (Chaverri et al. 2005a). Based on nucleotide comparisons, *R. sichuanensis* (SICAUCC 25-0188, culture ex-type) differs from *R. camerunensis* (ARSEF 7682, culture ex-type) by 7/770 bp (0.91%, 1 gap) in LSU, 18/732 bp (2.46%, without gap) in *rpb*1, and 11/960 bp (1.15%, without gap) in *tef*1-α, respectively. Consequently, based on combined morphological and molecular evidence, we propose *R. sichuanensis* as a new species.

## Discussion

Hypocrealean entomopathogenic fungi exhibit an exceptionally broad host range within *Insecta* and predominantly act as pathogens or parasites, establishing diverse ecological associations with various arthropods and non-arthropod microinvertebrates (Shrestha et al. 2016; Wei et al. 2023; Xiao et al. 2023). Notably, scale insects (*Hemiptera*, *Coccoidea*) represent a highly distinctive host group due to their specialized biological and morphological traits, including a sedentary lifestyle, permanently attached to plant surfaces, extreme reduction of legs and antennae in adult females, and the production of thick wax covers or sclerotized tests that serve as strong physical barriers against infection (Gullan and Kosztarab 1997; Normark 2003). Most scale insects are highly specialized plant parasites with limited dispersal ability, as only first-instar nymphs are mobile, while adults remain sessile throughout their lives (Hanks and Denno 1993). These unique features have driven the evolution of a specialized and phylogenetically distinct assemblage of hypocrealean entomopathogens that are highly adapted to infecting scale insects, thus representing a highly specialized distinct evolutionary guild (Chaverri et al. 2005a; Sung et al. 2007). Extensive studies on entomopathogenic fungi in Southwest China have uncovered numerous new species, particularly in Guizhou and Yunnan Provinces (Wang et al. 2020; Wei et al. 2023; Xiao et al. 2023; Chuang et al. 2024; Chen et al. 2025a, 2025b). However, research on scale insect-associated entomopathogenic fungi in Sichuan Province remains remarkably limited (Zha et al. 2019; Xu et al. 2021; Liu et al. 2023). This study provides a comprehensive taxonomic treatment of scale insect-associated entomopathogenic fungi using multi-locus phylogenetic inference combined with a detailed morphological examination. Our results reveal a diverse assemblage of nine scale insect-associated entomopathogenic fungi belonging to the genera *Conoideocrella*, *Hypocrella*, *Helicocollum*, and *Regiocrella* within *Clavicipitaceae (Hypocreales)*, all of which are obligate or specialized pathogens uniquely associated with scale insects. This research significantly advances current understanding of the morphological diversity, distribution, host specificity, and phylogenetic relationships of scale insect-associated entomopathogenic fungi within *Clavicipitaceae (Hypocreales)*.

The genus *Conoideocrella* was established by Johnson et al. (2009) to accommodate two distinct lineages of *Torrubiella* sensu lato, viz., *T. luteorostrata* Zimm. and *T. tenuis* Petch, based on morphological and phylogenetic analyses within *Clavicipitaceae (Hypocreales)*. To date, this genus comprises six species, viz., *C. luteorostrata* (Hywel-Jones 1993; Johnson et al. 2009; Lovett et al. 2022), *C. tenuis* (Johnson et al. 2009; Wang et al. 2024b), *C. krungchingensis* (Mongkolsamrit et al. 2016), *C. fenshuilingensis* (Wang et al. 2024b), *C. gongyashanensis* (Lin et al. 2025), and *C. tiankengensis* (Chen et al. 2025a), respectively (www.indexfungorum.org, accessed 25 March 2026). In this study, six *Conoideocrella* species associated with scale insects were identified, including two new species: *C. jiufengensis*, *C. violaceomarginata*, four new provincial distribution records: *C. luteorostrata*, *C. tenuis*, *C. fenshuilingensis*, *C. gongyashanensis*, and one taxonomic synonymization: *C. tiankengensis* is synonymized with *C. tenuis*. Additionally, except for *C. tenuis*, complete teleomorphic characters could not be observed in the other five species, owing to the immature developmental status of the available specimens. It is anticipated that these morphological features will be supplemented in future studies. Notably, most *Conoideocrella* species are entomopathogenic fungi that parasitize scale insects and produce a teleomorph, with the sole exception of *C. gongyashanensis*, which was discovered on dead spiders and for which only the anamorph has been described (Lin et al. 2025). *Conoideocrella* species are primarily characterized by elongated, conical perithecia, with interspecific differentiation occurring mainly based on stromatal color and shape (Hywel-Jones 1993; Johnson et al. 2009; Mongkolsamrit et al. 2016; Lovett et al. 2022; Wang et al. 2024b; Chen et al. 2025a; Lin et al. 2025). A key finding of this study is that phylogenetically basal species (i.e., *C. gongyashanensis*, *C. jiufengensis*, and *C. krungchingensis*; Fig. 1) produce only one or two conidia at the apex of the phialide, whereas other congeneric species form slimy conidial heads with multiple conidia at the phialide apex.

The genus *Hypocrella* was erected by Saccardo (1878) to accommodate four species initially classified in *Hypocrea* (*Hypocreaceae*, *Hypocreales*). Of these four species, only *Hypocrella discoidea* (Berk. & Broome) Sacc. has been retained as the type species of Hypocrella (Hywel-Jones and Evans 1993). Combining multi-locus phylogenetic analyses with morphological comparisons, Chaverri et al. (2008) greatly revised the generic limits of *Hypocrella*. Most taxa formerly classified under this genus were reallocated to the closely related genera *Moelleriella* and *Samuelsia*. Their phylogenetic framework further separated these three genera into discrete clades: *Moelleriella* was placed within the Effuse and Globose clades, *Hypocrella* within Pulvinate clade A, and *Samuelsia* within Pulvinate clade B (Chaverri et al. 2008). The new species *H. cinnamomum* reported herein is a member of Pulvinate clade A. The genus *Aschersonia* was established by Montagne (1848), with *A. tahitensis* Mont. as type species. Anamorphs of *Hypocrella* are classified in the anamorph genus *Aschersonia* (Chaverri et al. 2005b). Following the nomenclatural proposal of Rossman et al. (2016), *Hypocrella* has been formally conserved and given precedence over its synonym *Aschersonia*, owing to its wide recognition and greater species diversity. *Hypocrella* is characterized by brightly colored, discoid to pulvinate stromata, globose to pyriform perithecia, filiform ascospores, pycnidium-like conidiomata, and fusiform conidia (Chaverri et al. 2008; Mongkolsamrit et al. 2009; Crous et al. 2025; Wang et al. 2025b). These fungi are primarily distributed in tropical regions, with a few species occurring in subtropical and temperate zones (Saccardo 1878; Hywel-Jones and Evans 1993; Chaverri et al. 2005b; Chaverri et al. 2008; Mongkolsamrit et al. 2009; He et al. 2017). They can mass-infect scale insects and whiteflies parasitizing living leaves or twigs, inducing epizootics in these hosts. This phenomenon is particularly evident in humid tropical forests. Species of the genus *Hypocrella* have potential as biological control agents. For instance, *H. discoidea* (Berk. & Broome) Sacc. and *H. siamensis* Hywel-Jones & Mongkols. are recognized as promising candidate biological control strains for the management of scale insects (Liu et al. 2006; Mongkolsamrit et al. 2009; Agrawal et al. 2016; Luangsa-ard et al. 2026). In addition, reports on the insecticidal properties of metabolites produced by *Hypocrella* fungi are increasing, and their natural products exhibit diverse biological activities, including insecticidal, antibacterial, antimalarial, anticancer, antiviral, and anti-inflammatory effects (Isaka et al. 2005, 2011; Guo et al. 2015).

The genus *Helicocollum* was originally established by Luangsa-ard et al. (2017), with *He. surathaniensis* Luangsa-ard, Mongkols., Noisrip. & Thanakitp. designated as the type species. Members of the genus are morphologically characterized by pulvinate to planar stromata, and pyriform, lanceolate or narrowly lanceolate phialides, tapering gradually into a helical neck, and cylindrical or fusiform conidia, 1–2-septate. To date, four species have been described in *Helicocollum*, all of which were originally reported from scale insects in Thailand (www.indexfungorum.org, accessed 25 March 2026). *He. krabiense* Luangsa-ard, Mongkols., Noisrip. & Thanakitp., *He. samlanense* Mongkols., Noisrip. & Luangsa-ard, and *He. surathaniense* occur on the abaxial surfaces of bamboo leaves, whereas *He. chanthaburiense* Luangsa-ard, Mongkols., Noisrip. & Thanakitp. is found on the underside of leaves *Durio zibethinus* L (Luangsa-ard et al. 2017; Crous et al. 2024). Among these taxa, only *He. samlanense* possesses confirmed teleomorphic structures, while the remaining species are currently known exclusively by their anamorphic stages (Luangsa-ard et al. 2017; Crous et al. 2024). In the present study, *He. bambusae* was identified from scale insects on the abaxial surfaces of bamboo leaves in Sichuan Province, China, and only its anamorph was observed. This discovery extends the known distribution of the genus from tropical regions to subtropical areas. It also provides further evidence that members of *Helicocollum* preferentially colonize scale insects on the abaxial surfaces of bamboo leaves.

*Regiocrella* was established by Chaverri et al. (2005a) based on specimens collected from Cameroon and China, with *R. camerunensis* P. Chaverri & H.C. Evans designated as the type species, *R. sinensis* P. Chaverri & K.T. Hodge concomitantly described as the second species of the genus. Both species are obligately parasitic on scale insects, but show no association with host plants. The genus is characterized by perithecia partially immersed in the subiculum, non-capitate asci, unicellular fusiform ascospores, and conidiophores forming a pycnidial-acervular conidiomatal complex. All species produce mucilaginous conidial masses at the apex of phialides. This trait represents an adaptive evolutionary feature, facilitating dispersal by *Hymenoptera* and *Formicidae* that feed on scale insect honeydew, as well as short-distance dispersal through rain splash (Chaverri and Samuels 2003; Chaverri et al. 2005a). This dispersal strategy constitutes a core evolutionary adaptation of *Regiocrella* to the microhabitat of its scale insect hosts (Chaverri et al. 2005a).

## Conclusion

This study performed a polyphasic taxonomic investigation of scale insect-associated entomopathogenic fungi in Sichuan Province, China, using multi-locus phylogeny and morphology. We documented nine species in four genera: *Conoideocrella*, *Hypocrella*, *Helicocollum*, and *Regiocrella*, including five new species, four new provincial records, and one new synonym. Both anamorphic and teleomorphic states of *C. fenshuilingensis* and *C. gongyashanensis* are described simultaneously for the first time, with expanded host and geographic distributions for several *Conoideocrella* species. Phylogenetic analyses revealed a key evolutionary pattern in basal *Conoideocrella* lineages, which produce 1–2 conidia at the phialide apex, unlike other congeners with slimy conidial heads. The discovery of *Helicocollum bambusae* extends the genus into subtropical China and supports its host preference for bamboo-infesting scale insects. These findings advance our understanding of the diversity, distribution, and morphological evolution of entomopathogenic fungi within *Clavicipitaceae* in Southwest China, and provide a basis for future research on ecological adaptation and biocontrol potential. Further studies on host specificity and agricultural applications are recommended.

## Supplementary Material

XML Treatment for Conoideocrella fenshuilingensis

XML Treatment for Conoideocrella gongyashanensis

XML Treatment for Conoideocrella jiufengensis

XML Treatment for Conoideocrella luteorostrata

XML Treatment for Conoideocrella tenuis

XML Treatment for Conoideocrella violaceomarginata

XML Treatment for Hypocrella cinnamomum

XML Treatment for Helicocollum bambusae

XML Treatment for Regiocrella sichuanensis

## Additional information

### Artificial Intelligence (AI) use

The authors accept full responsibility for the content of the manuscript, including the disclosure of any use of AI.

Regarding the use of AI in the preparation of this manuscript, the authors declare the following: DeepSeek

Used for: Language, style and writing

AI was used for correcting English grammar and style only.

### Adherence to national and international regulations

All the fungal strains used in this study have been legally obtained, respecting the Convention on Biological Diversity (Rio Convention).

### Author ORCIDs

F. Liu https://orcid.org/0000-0003-4580-7169

F. Wang https://orcid.org/0000-0002-3227-5194

X. Xu https://orcid.org/0000-0002-6832-5421

S. Xiang https://orcid.org/0009-0001-3617-3427

X. Li https://orcid.org/0009-0006-5189-0922

H. Yang https://orcid.org/0000-0002-7523-5862

S. Li https://orcid.org/0000-0003-3328-5332

C. Yang https://orcid.org/0000-0002-3854-8835

### Data availability

All sequence data generated for this study can be accessed via GenBank (https://www.ncbi.nlm.nih.gov/genbank/).

## Supplementary materials

Supplementary material 1 RPB1-mafft Data type fasta https://binary.pensoft.net/file/1691476This dataset is made available under the Open Database License (http://opendatacommons.org/licenses/odbl/1.0/). The Open Database License (ODbL) is a license agreement intended to allow users to freely share, modify, and use this Dataset while maintaining this same freedom for others, provided that the original source and author(s) are credited. Feng Liu

Supplementary material 2 Concatenation-LSU-*tef*1-α-*rpb*1 Data type fas https://binary.pensoft.net/file/1691477This dataset is made available under the Open Database License (http://opendatacommons.org/licenses/odbl/1.0/). The Open Database License (ODbL) is a license agreement intended to allow users to freely share, modify, and use this Dataset while maintaining this same freedom for others, provided that the original source and author(s) are credited. Feng Liu

Supplementary material 3 TEF-mafft Data type fasta https://binary.pensoft.net/file/1691478This dataset is made available under the Open Database License (http://opendatacommons.org/licenses/odbl/1.0/). The Open Database License (ODbL) is a license agreement intended to allow users to freely share, modify, and use this Dataset while maintaining this same freedom for others, provided that the original source and author(s) are credited. Feng Liu

Supplementary material 4 LSU-mafft Data type fasta https://binary.pensoft.net/file/1691479This dataset is made available under the Open Database License (http://opendatacommons.org/licenses/odbl/1.0/). The Open Database License (ODbL) is a license agreement intended to allow users to freely share, modify, and use this Dataset while maintaining this same freedom for others, provided that the original source and author(s) are credited. Feng Liu

Supplementary material 5 Phylogenetic tree on LSU Data type pdf Explanation note Maximum likelihood (RAxML) tree derived from the LSU dataset depicting relationships within Clavicipitaceae and allied genera. Nodes are supported by ML bootstrap values (MLBS ≥ 60%) and Bayesian posterior probabilities (BPP ≥ 0.90), shown as MLBS/BPP. https://binary.pensoft.net/file/1691480This dataset is made available under the Open Database License (http://opendatacommons.org/licenses/odbl/1.0/). The Open Database License (ODbL) is a license agreement intended to allow users to freely share, modify, and use this Dataset while maintaining this same freedom for others, provided that the original source and author(s) are credited. Feng Liu

Supplementary material 6 Phylogenetic tree on *tef*1-α Data type pdf Explanation note Maximum likelihood (RAxML) tree derived from the tef1-α dataset depicting relationships within Clavicipitaceae and allied genera. Nodes are supported by ML bootstrap values (MLBS ≥ 60%) and Bayesian posterior probabilities (BPP ≥ 0.90), shown as MLBS/BPP. https://binary.pensoft.net/file/1691481This dataset is made available under the Open Database License (http://opendatacommons.org/licenses/odbl/1.0/). The Open Database License (ODbL) is a license agreement intended to allow users to freely share, modify, and use this Dataset while maintaining this same freedom for others, provided that the original source and author(s) are credited. Feng Liu

Supplementary material 7 Phylogenetic tree on *rpb*1 Data type pdf Explanation note Maximum likelihood (RAxML) tree derived from the rpb1 dataset depicting relationships within Clavicipitaceae and allied genera. Nodes are supported by ML bootstrap values (MLBS ≥ 60%) and Bayesian posterior probabilities (BPP ≥ 0.90), shown as MLBS/BPP. https://binary.pensoft.net/file/1691482This dataset is made available under the Open Database License (http://opendatacommons.org/licenses/odbl/1.0/). The Open Database License (ODbL) is a license agreement intended to allow users to freely share, modify, and use this Dataset while maintaining this same freedom for others, provided that the original source and author(s) are credited. Feng Liu

## References

[B1] Agrawal Y, Narwani T, Subramanian S (2016) Genome sequence and comparative analysis of clavicipitaceous insect-pathogenic fungus *Aschersonia badia* with *Metarhizium* spp. BMC Genomics 17(1): 367. 10.1186/s12864-016-2710-6 PMC4869207 27189621

[B2] Araújo JPM, Hughes DP (2016) Diversity of entomopathogenic fungi: which groups conquered the insect body? Advances in Genetics 94: 1–39. 10.1101/003756 27131321

[B3] Ashraf SA, Elkhalifa AEO, Siddiqui AJ et al. (2020) Cordycepin for health and wellbeing: a potent bioactive metabolite of an entomopathogenic medicinal fungus *Cordyceps* with its nutraceutical and therapeutic potential. Molecules 25(12): 2735. 10.3390/molecules25122735 PMC7356751 32545666

[B4] Behie SW, Bidochka MJ (2014) Ubiquity of insect-derived nitrogen transfer to plants by endophytic insect-pathogenic fungi: an additional branch of the soil nitrogen cycle. Applied and Environmental Microbiology 80(5): 1553–1560. 10.1128/AEM.03338-13 PMC3957595 24334669

[B5] Bihal R, Al-Khayri JM, Banu AN et al. (2023) Entomopathogenic fungi: an eco-friendly synthesis of sustainable nanoparticles and their nanopesticide properties. Microorganisms 11(6): 1617. 10.3390/microorganisms11061617 PMC10302739 37375119

[B6] Bischoff JF, Rehner SA, Humber RA (2009) A multilocus phylogeny of the *Metarhizium anisopliae* lineage. Mycologia 101(4): 512–530. 10.3852/07-202 19623931

[B7] Castlebury LA, Rossman AY, Sung GH et al. (2004) Multigene phylogeny reveals new lineage for *Stachybotrys chartarum*, the indoor air fungus. Mycological Research 108(8): 864–872. 10.1017/S0953756204000607 15449591

[B8] Chaverri P, Bischoff JF, Evans HC et al. (2005a) *Regiocrella*, a new entomopathogenic genus with a pycnidial anamorph and its phylogenetic placement in the *Clavicipitaceae*. Mycologia 97(6): 1225–1237. 10.1080/15572536.2006.11832732 16722216

[B9] Chaverri P, Bischoff JF, Liu M et al. (2005b) A new species of *Hypocrella*, *H. macrostroma*, and its phylogenetic relationships to other species with large stromata. Mycological Research 109(11): 1268–1275. 10.1017/s0953756205003904 16279420

[B10] Chaverri P, Liu M, Hodge KT (2008) A monograph of the entomopathogenic genera *Hypocrella*, *Moelleriella*, and *Samuelsia* gen. nov. (*Ascomycota*, *Hypocreales*, *Clavicipitaceae*), and their aschersonia-like anamorphs in the Neotropics. Studies in Mycology 60: 1–66. 10.3114/sim.2008.60.01 PMC2275321 18490956

[B11] Chaverri P, Samuels GJ (2003) *Hypocrea*/*Trichoderma* (*Ascomycota*, *Hypocreales*, *Hypocreaceae*): species with green ascospores. Studies in Mycology 48(48): 1–116.

[B12] Chen WH, Shu HL, Li D et al. (2025a) Morphological and phylogenetic evidence reveals three new arthropod-associated species of *Hypocreales* (*Clavicipitaceae*, *Bionectriaceae*, and *Myrotheciomycetaceae*) from karst habitats in Guizhou, China. MycoKeys 123: 319–353. 10.3897/mycokeys.123.164334 PMC12552844 41140783

[B13] Chen WH, Shu HL, Li D et al. (2025b) Shedding light on the darkness: cryptic diversity of cordyceps-like fungi in karst regions of Guizhou Province, China. Mycosphere 16(1): 2887–2974. 10.5943/mycosphere/16/1/20

[B14] Chen YX, Xue QL, Xie YX et al. (2020) *Moelleriella sinensis* sp. nov. (*Clavicipitaceae*, *Ascomycota*) from Southeast China. Phytotaxa 429(4): 22. 10.11646/phytotaxa.429.4.5

[B15] Chethana KWT, Manawasinghe IS, Hurdeal VG et al. (2021) What are fungal species and how to delineate them? Fungal diversity 109(1): 1–25. 10.1007/s13225-021-00483-9

[B16] Chuang WY, Lin YC, Shrestha B et al. (2024) Phylogenetic diversity and morphological characterization of cordycipitaceous species in Taiwan. Studies in Mycology 109: 1–56. 10.3114/sim.2024.109.01 PMC11663429 39717658

[B17] Crous PW, Catcheside DEA, Catcheside PS et al. (2025) Fungal planet description sheets: 1781–1866. Persoonia 54(1): 327–587. 10.3114/persoonia.2025.54.10 PMC12308287 40746709

[B18] Crous PW, Jurjević Ž, Balashov S et al. (2024) Fungal planet description sheets: 1614–1696. Fungal Systematics and Evolution 13(1): 183–440. 10.3114/fuse.2024.13.11 PMC11320056 39140100

[B19] De Wint FC, Nicholson S, Koid QQ et al. (2024) Introducing a global database of entomopathogenic fungi and their host associations. Scientific Data 11(1): 1418. 10.1038/s41597-024-04103-4 PMC11663221 39709508

[B20] Ferron P (1978) Biological control of insect pests by entomogenous fungi. Annual Review of Entomology 23(1): 409–442. 10.1146/annurev.en.23.010178.002205

[B21] Gielen R, Ude K, Kaasik A et al. (2024) Entomopathogenic fungi as mortality agents in insect populations: a review. Ecology and Evolution 14(12): e70666. 10.1002/ece3.70666 PMC11620982 39650537

[B22] Gullan PJ, Kosztarab M (1997) Adaptations in scale insects. Annual Review of Entomology 42(1): 23–50. 10.1146/annurev.ento.42.1.23 15012306

[B23] Guo QF, Dong LL, Zang XY et al. (2015) A new azaphilone from the entomopathogenic fungus *Hypocrella* sp. 29: 2000–2006. 10.1080/14786419.2015.1023199 25801461

[B24] Gurulingappa P, McGee PA, Sword G (2011) Endophytic *Lecanicillium lecanii* and *Beauveria bassiana* reduce the survival and fecundity of *Aphis gossypii* following contact with conidia and secondary metabolites. Crop Protection 30(3): 349–353. 10.1016/j.cropro.2010.11.017

[B25] Hall TA (2011) BioEdit: an important software for molecular biology. GERF Bulletin of Biosciences 2: 60–61.

[B26] Hanks LM, Denno RF (1993) Natural enemies and plant water relations influence the distribution of an armored scale insect. Ecology 74(4): 1081–1091. 10.2307/1940478

[B27] He XY, Guo QF, Fu DL et al. (2017) Isolation, identification and antitumor activities of natural products from *Hypocrella* sp. Mycosystema 36(5): 604–610. 10.13346/j.mycosystema.170040

[B28] Hou LW, Giraldo A, Groenewald JZ et al. (2023) Redisposition of acremonium-like fungi in *Hypocreales*. Studies in Mycology 105: 23–203. 10.3114/sim.2023.105.02 PMC11182610 38895703

[B29] Humber RA (2008) Evolution of entomopathogenicity in fungi. Journal of Invertebrate Pathology 98(3): 262–266. 10.1016/j.jip.2008.02.017 18423482

[B30] Hyde KD, Baldrian P, Chen YP et al. (2024a) Current trends, limitations and future research in the fungi? Fungal Diversity 125(1): 1–71. 10.1007/s13225-023-00532-5

[B31] Hyde KD, Noorabadi MT, Thiyagaraja V et al. (2024b) The 2024 Outline of Fungi and fungus-like taxa. Mycosphere 15(1): 5146–6239. 10.5943/mycosphere/15/1/25

[B32] Hyde KD, Xu JC, Rapior S et al. (2019) The amazing potential of fungi: 50 ways we can exploit fungi industrially. Fungal Diversity 97(1): 1–136. 10.1007/s13225-019-00430-9

[B33] Hywel-Jones NL (1993) *Torrubiella luteorostrata*: a pathogen of scale insects and its association with *Paecilomyces cinnamomeus* with a note on *Torrubiella tenuis*. Mycological Research 97: 1126–1130. 10.1016/S0953-7562(09)80514-5

[B34] Hywel-Jones NL, Evans HC (1993) Taxonomy and ecology of *Hypocrella discoidea* and its anamorph, *Aschersonia samoensis*. Mycological Research 97(7): 871–876. 10.1016/s0953-7562(09)81165-9

[B35] Isaka M, Chinthanom P, Sappan M et al. (2011) Lanostane and hopane triterpenes from the entomopathogenic fungus *Hypocrella* sp. BCC 14524. Journal of Natural Products 74(10): 2143–2150. 10.1021/np200429b 21995505

[B36] Isaka M, Palasarn S, Kocharin K et al. (2005) A cytotoxic xanthone dimer from the entomopathogenic fungus *Aschersonia* sp. BCC 8401. Journal of Natural Products 68(6): 945–946. 10.1021/np058028h 15974626

[B37] Jeewon R, Hyde KD (2016) Establishing species boundaries and new taxa among fungi: recommendations to resolve taxonomic ambiguities. Mycosphere 7(11): 1669–1677. 10.5943/mycosphere/7/11/4

[B38] Johnson D, Sung GH, Hywel-Jones NL et al. (2009) Systematics and evolution of the genus *Torrubiella* (*Hypocreales*, *Ascomycota*). Mycological Research 113(3): 279–289. 10.1016/j.mycres.2008.09.008 18938242

[B39] Kabaluk JT, Ericsson JD (2007) *Metarhizium anisopliae* seed treatment increases yield of field corn when applied for wireworm control. Agronomy Journal 99(5): 1377–1381. 10.2134/agronj2007.0017N

[B40] Kankanamalage HPA, Yang JY, Karunarathna SC et al. (2025) Entomopathogenic fungi: insights into recent understanding. World Journal of Microbiology and Biotechnology 41(6): 179. 10.1007/s11274-025-04377-9 40415063

[B41] Katoh K, Rozewicki J, Yamada KD (2019) MAFFT online service: multiple sequence alignment, interactive sequence choice and visualization. Briefings in Bioinformatics 20(4): 1160–1166. 10.1093/bib/bbx108 PMC6781576 28968734

[B42] Kepler RM, Sung GH, Harada Y et al. (2012) Host jumping onto close relatives and across kingdoms by *Tyrannicordyceps (Clavicipitaceae)* gen. nov. and *Ustilaginoidea (Clavicipitaceae)*. American Journal of Botany 99(3): 552–561. 10.3732/ajb.1100124 22334447

[B43] Kerry BR (2000) Rhizosphere interactions and the exploitation of microbial agents for the biological control of plant-parasitic nematodes. Annual Review of Phytopathology 38: 423–441. 10.1146/annurev.phyto.38.1.423 11701849

[B44] Lin LB, Lin YS, Keyhani NO et al. (2025) New entomopathogenic species in the *Clavicipitaceae* family (*Hypocreales*, *Ascomycota*) from the subtropical forests of Fujian, China. Frontiers in Microbiology 16: 423. 10.3389/fmicb.2025.1532341 PMC11911381 40099183

[B45] Litwin A, Nowak M, Różalska S (2020) Entomopathogenic fungi: unconventional applications. Reviews in Environmental Science and Bio/Technology 19(1): 23–42. 10.1007/s11157-020-09525-1

[B46] Liu F, Deng Y, Wang FH et al. (2023) Morphological and molecular analyses reveal two new species of *Microcera* (*Nectriaceae*, *Hypocreales*) associated with scale insects on walnut in China. MycoKeys 98: 19–35. 10.3897/mycokeys.98.103484 PMC10242524 37287767

[B47] Liu M, Chaverri P, Hodge KT (2006) A taxonomic revision of the insect biocontrol fungus *Aschersonia aleyrodis*, its allies with white stromata and their *Hypocrella* sexual states. Mycological Research 110(5): 537–554. 10.1016/j.mycres.2006.01.013 16769508

[B48] Lovett B, Barrett H, Macias AM et al. (2022) Morphological and phylogenetic resolution of *Conoideocrella luteorostrata* (*Hypocreales*: *Clavicipitaceae*), a potential biocontrol fungus for *Fiorinia externa* in United States Christmas tree production areas. Mycologia 116(2): 267–290. 10.1101/2022.10.18.512709 38275281

[B49] Luangsa-ard J, Houbraken J, van Doorn T et al. (2011) *Purpureocillium*, a new genus for the medically important *Paecilomyces lilacinus*. FEMS Microbiology Letters 321(2): 141–149. 10.1111/j.1574-6968.2011.02322.x 21631575

[B50] Luangsa-ard J, Tasanathai K, Thanakitpipattana D et al. (2018) Novel and interesting *Ophiocordyceps* spp. (*Ophiocordycipitaceae*, *Hypocreales*) with superficial perithecia from Thailand. Studies in Mycology 89: 125–142. 10.1016/j.simyco.2018.02.001 PMC6002337 29910519

[B51] Luangsa-ard JJ, Mongkolsamrit S, Noisripoom W et al. (2017) *Helicocollum*, a new clavicipitalean genus pathogenic to scale insects (*Hemiptera*) in Thailand. Mycological Progress 16(4): 419–431. 10.1007/s11557-017-1283-3

[B52] Luangsa-ard JJ, Thanakitpipattana D, Mongkolsamrit S et al. (2026) Atlas of hypocrealean invertebrate-pathogenic fungi of Thailand. Studies in Mycology 114: 1–369. https://10.3114/sim.2026.114.01 10.3114/sim.2026.114.01 PMC13482074 42614492

[B53] Mains EB (1948) Entomogenous fungi. Mycologia 40(4): 402–416. 10.1080/00275514.1944.12017718

[B54] Miller MA, Pfeiffer W, Schwartz T (2010) Creating the CIPRES science gateway for inference of large phylogenetic tree, in Proceedings of the Gateway Computing Environments Workshop (GCE), 14 November 2010, New Orleans, 1–8. 10.1109/GCE.2010.5676129

[B55] Mongkolsamrit S, Khonsanit A, Thanakitpipattana D et al. (2020) Revisiting *Metarhizium* and the description of new species from Thailand. Studies in Mycology 95: 171–251. 10.1016/j.simyco.2020.04.001 PMC7426330 32855740

[B56] Mongkolsamrit S, Luangsa-ard JJ, Hywel-Jones NL (2011) *Samuelsia mundiveteris* sp. nov. from Thailand. Mycologia 103(4): 921–927. 10.3852/11-049 21482629

[B57] Mongkolsamrit S, Luangsa-ard JJ, Spatafora JW et al. (2009) A combined ITS rDNA and β-tubulin phylogeny of Thai species of *Hypocrella* with non-fragmenting ascospores. Mycological Research 113(6–7): 684–699. 10.1016/j.mycres.2009.02.004 19249367

[B58] Mongkolsamrit S, Noisripoom W, Thanakitpipattana D et al. (2018) Disentangling cryptic species with isaria-like morphs in *Cordycipitaceae*. Mycologia 110(1): 230–257. 10.1080/00275514.2018.1446651 29863995

[B59] Mongkolsamrit S, Thanakitpipattana D, Khonsanit A et al. (2016) *Conoideocrella krungchingensis* sp. nov., an entomopathogenic fungus from Thailand. Mycoscience 57(4): 264–270. 10.1016/j.myc.2016.03.003

[B60] Montagne JPFC (1848) Sixième centurie de plantes cellulaires exotiques nouvelles. Cryptogamae Taitenses. Annales des Sciences Naturelles Botanique, Série 3, 10: 106–136.

[B61] Nicoletti R, Becchimanzi A (2020) Endophytism of *Lecanicillium* and *Akanthomyces*. Agriculture 10(6): 205. 10.3390/agriculture10060205

[B62] Nishi O, Sushida H, Higashi Y et al. (2020) Epiphytic and endophytic colonisation of tomato plants by the entomopathogenic fungus *Beauveria bassiana* strain GHA. International Biomechanics 12(1): 39–47. 10.1080/21501203.2019.1707723 PMC7889226 33628607

[B63] Nishi O, Sushida H, Higashi Y et al. (2022) Entomopathogenic fungus *Akanthomyces muscarius* (*Hypocreales*: *Cordycipitaceae*) strain IMI 268317 colonises on tomato leaf surface through conidial adhesion and general and microcycle conidiation. Mycology 13(2): 133–142. 10.1080/21501203.2021.1944929 PMC9196654 35711329

[B64] Normark BB (2003) The evolution of alternative genetic systems in scale insects. Annual Review of Entomology 48(1): 397–423. 10.1146/annurev.ento.48.091801.112703 12221039

[B65] Ownley BH, Gwinn KD, Vega FE (2010) Endophytic fungal entomopathogens with activity against plant pathogens: ecology and evolution. BioControl 55(1): 113–128. 10.1007/s10526-009-9241-x

[B66] Perera RH, Hyde KD, Jones EBG et al. (2023) Profile of *Bionectriaceae*, *Calcarisporiaceae*, *Hypocreaceae*, *Nectriaceae*, *Tilachlidiaceae*, *Ijuhyaceae* fam. nov., *Stromatonectriaceae* fam. nov. and *Xanthonectriaceae* fam. nov. Fungal Diversity 118(1): 95–271. 10.1007/s13225-022-00512-1

[B67] Que SQ, Yu AL, Liu YJ et al. (2019) Application research progress of *Beauveria*. Forest Pest and Disease 38(2): 29–35. 10.3969/j.issn.1671-0886.2019.02.007

[B68] Rehner SA, Buckley E (2005) A *Beauveria* phylogeny inferred from nuclear ITS and EF1-alpha sequences: evidence for cryptic diversification and links to *Cordyceps* teleomorphs. Mycologia 97(1): 84–98. 10.3852/mycologia.97.1.84 16389960

[B69] Rehner SA, Samuels GJ (1995) Molecular systematics of the *Hypocreales*: a teleomorph gene phylogeny and the status of their anamorphs. Canadian Journal of Botany 73(S1): 816–823. 10.1139/b95-327

[B70] Ríos-Moreno A, Garrido-Jurado I, Resquín-Romero G et al. (2016) Destruxin a production by *Metarhizium brunneum* strains during transient endophytic colonisation of *Solanum tuberosum*. Biocontrol Science and Technology 26(11): 1574–1585. 10.1080/09583157.2016.1223274

[B71] Rossman AY (1996) Morphological and molecular perspectives on systematics of the *Hypocreales*. Mycologia 88(1): 1–19. 10.1080/00275514.1996.12026620

[B72] Rossman AY, Allen WC, Braun U et al. (2016) Overlooked competing asexual and sexually typified generic names of *Ascomycota* with recommendations for their use or protection. IMA Fungus 7: 289–308. 10.5598/imafungus.2016.07.02.09 PMC5159600 27990336

[B73] Saccardo PA (1878) Enumeratio pyrenomycetum hypocreaceorum hucusque cognitorum systemate carpologico dispositorum. Michelia 1(3): 277–325. 10.5962/bhl.title.5371

[B74] Schardl CL, Young CA, Hesse U et al. (2013) Plant-symbiotic fungi as chemical engineers: multi-genome analysis of the *Clavicipitaceae* reveals dynamics of alkaloid loci. PLoS Genetics 9(2): e1003323. 10.1371/journal.pgen.1003323 PMC3585121 23468653

[B75] Shrestha B, Tanaka E, Hyun MW et al. (2016) Coleopteran and lepidopteran hosts of the entomopathogenic genus *Cordyceps* sensu lato. Journal of Mycology 2016: 1–14. 10.1155/2016/7648219

[B76] Spatafora JW, Sung GH, Sung JM et al. (2007) Phylogenetic evidence for an animal pathogen origin of ergot and the grass endophytes. Molecular Ecology 16(8): 1701–1711. 10.1111/j.1365-294x.2007.03225.x 17402984

[B77] St. Leger RJ, Wang C (2010) Genetic engineering of fungal biocontrol agents to achieve greater efficacy against insect pests. Applied Microbiology and Biotechnology 85(4): 901–907. 10.1007/s00253-009-2306-z 19862514

[B78] Sullivan RF, Bills GF, Hywel-Jones NL et al. (2000) *Hyperdermium*: a new clavicipitalean genus for some tropical epibionts of dicotyledonous plants. Mycologia 92(5): 908–918. 10.1080/00275514.2000.12061236

[B79] Sung GH, Sung JM, Hywel-Jones NL et al. (2007) A multi-gene phylogeny of *Clavicipitaceae* (*Ascomycota*, Fungi): Identification of localized incongruence using a combinational bootstrap approach. Molecular Phylogenetics and Evolution 44(3): 1204–1223. 10.1016/j.ympev.2007.03.011 17555990

[B80] Tang DX, Xu ZH, Wang Y et al. (2023) Multigene phylogeny and morphology reveal two novel zombie-ant fungi in *Ophiocordyceps* (*Ophiocordycipitaceae*, *Hypocreales*). Mycological Progress 22(4): 1–22. 10.1007/s11557-023-01874-9

[B81] Thanakitpipattana D, Tasanathai K, Mongkolsamrit S et al. (2020) Fungal pathogens occurring on *Orthopterida* in Thailand. Persoonia 44(1): 140–160. 10.3767/persoonia.2020.44.06 PMC7567961 33116339

[B82] Vaidya G, Lohman DJ, Meier R (2011) SequenceMatrix: concatenation software for the fast assembly of multi-gene datasets with character set and codon information. Cladistics 27(2): 171–180. 10.1111/j.1096-0031.2010.00329.x 34875773

[B83] Vega FE, Meyling NV, Luangsa-ard JJ et al. (2012) Fungal entomopathogens. Insect Pathology 2: 171–220. 10.1016/B978-0-12-384984-7.00006-3

[B84] Vilgalys R, Hester M (1990) Rapid genetic identification and mapping of enzymatically amplified ribosomal DNA from several *Cryptococcus* species. Journal of Bacteriology 172(8): 4238–4246. 10.1128/jb.172.8.4238-4246.1990 PMC213247 2376561

[B85] Wang K, Cai L (2022) Annual review on nomenclature novelties of fungi in the world (2021). Biodiversity Science 30(08): 78–87. 10.17520/biods.2022277

[B86] Wang K, Cai L (2023) Annual review on nomenclature novelties of fungi in the world (2022). Biodiversity Science 31(10): 67–75. 10.17520/biods.2023176

[B87] Wang K, Cai L, Yao YJ (2021) Annual review on nomenclature novelties of fungi in China and the world (2020). Biodiversity Science 29(08): 1064–1072. 10.17520/biods.2021202

[B88] Wang K, Zhao MJ, Cai L (2024a) Annual review on fungi nomenclature novelties in China and around the world (2023). Biodiversity Science 32(11): 188–197. 10.17520/biods.2024361

[B89] Wang K, Zhao MJ, Cai L (2025a) Annual review on nomenclature novelties of fungi in China and around the world (2024). Biodiversity Science 33(10): 57–65. 10.17520/biods.2025355

[B90] Wang YB, Wang Y, Fan Q et al. (2020) Multigene phylogeny of the family *Cordycipitaceae (Hypocreales)*: new taxa and the new systematic position of the Chinese cordycipitoid fungus *Paecilomyces hepiali*. Fungal Diversity 103(1): 1–46. 10.1007/s13225-020-00457-3

[B91] Wang ZQ, Ma JM, Yang ZL et al. (2024b) Morphological and phylogenetic analyses reveal three new species of entomopathogenic fungi belonging to *Clavicipitaceae* (*Hypocreales*, *Ascomycota*). Journal of Fungi 10(6): 423. 10.3390/jof10060423 PMC11204714 38921409

[B92] Wang ZQ, Yang ZL, Zhao J et al. (2025b) Taxonomy and phylogeny of entomopathogenic fungi from China–revealing two new genera and thirteen new species within *Clavicipitaceae* (*Hypocreales*, *Ascomycota*). MycoKeys 117: 121–169. 10.3897/mycokeys.117.140577 PMC12070072 40364896

[B93] Wei DP, Gentekaki E, Wanasinghe DN et al. (2023) Diversity, molecular dating and ancestral characters state reconstruction of entomopathogenic fungi in *Hypocreales*. Mycosphere 13(1): 281–351. 10.5943/mycosphere/si/1f/8

[B94] Wei DP, Gentekaki E, Luangsa-ard JJ et al. (2026) Taxonomy, phylogeny and diversity of hyperparasites and their allied entomopathogens in *Hypocreales*. Studies in Mycology 114: 370–436. 10.3114/sim.2026.114.02 PMC13487413 42621338

[B95] Wijayawardene NN, Hyde KD, Dai DQ et al. (2022) Outline of fungi and fungus-like taxa–2021. Mycosphere 13(1): 53–453. 10.5943/mycosphere/13/1/2

[B96] Xiao YP, Wang YB, Hyde KD et al. (2023) *Polycephalomycetaceae*, a new family of clavicipitoid fungi segregates from *Ophiocordycipitaceae*. Fungal Diversity 120(1): 1–76. 10.1007/s13225-023-00517-4

[B97] Xu XL, Zeng Q, Lv YC et al. (2021) Insight into the systematics of novel entomopathogenic fungi associated with armored scale insect, *Kuwanaspis howardi* (*Hemiptera*: *Diaspididae*) in China. Journal of Fungi 7(8): 628. 10.3390/jof7080628 PMC8401669 34436167

[B98] Xu Y, Shen ZH, Ying LX et al. (2017) Hotspot analyses indicate significant conservation gaps for evergreen broadleaved woody plants in China. Scientific Reports 7(1): 1859. 10.1038/s41598-017-02098-0 PMC5431964 28500284

[B99] Yang CL, Xu XL, Li XY et al. (2025) Morphological and phylogenetic analyses reveal novel entomopathogenic fungi infecting scale insects and aphids in China. IMA Fungus 16: e170123. 10.3897/imafungus.16.170123 PMC12529078 41112861

[B100] Yang J, Liu LL, Jones EBG et al. (2023) Freshwater fungi from karst landscapes in China and Thailand. Fungal Diversity 119(1): 1–212. 10.1007/s13225-023-00514-7

[B101] Yuan HS, Lu X, Dai YC et al. (2020) Fungal diversity notes 1277–1386: taxonomic and phylogenetic contributions to fungal taxa. Fungal Diversity 104(1): 1–266. 10.1007/s13225-020-00461-7

[B102] Zha LS, Wen TC, Huang SK et al. (2019) Taxonomy and biology of *Cordyceps qingchengensis* sp. nov. and its allies. Phytotaxa 416(1): 14–24. 10.11646/phytotaxa.416.1.2

[B103] Zhang LW, Fasoyin OE, Molnár I et al. (2020a) Secondary metabolites from hypocrealean entomopathogenic fungi: novel bioactive compounds. Natural Product Reports 37(9): 1181–1206. 10.1039/c9np00065h PMC7529686 32211639

[B104] Zhang LW, Yue Q, Wang C et al. (2020b) Secondary metabolites from hypocrealean entomopathogenic fungi: genomics as a tool to elucidate the encoded parvome. Natural Product Reports 37(9): 1164–1180. 10.1039/d0np00007h PMC7529689 32211677

[B105] Zhao L, Groenewald JZ, Hou LW et al. (2026) New insights into acremonium-like fungi in *Hypocreales*: A taxonomic and phylogenetic perspective. Studies in Mycology 113: 1–71. 10.3114/sim.2026.113.01 PMC13081845 41993082

