# Supplementary figures and images for: Polyphasic taxonomy of entomopathogenic fungi infecting scale insects in Clavicipitaceae (Hypocreales) from China
Source: IMA Fungus. 2026 Jun 23;17:e195753. doi: 10.3897/imafungus.17.195753 (PMC13320307; doi:10.3897/imafungus.17.195753)

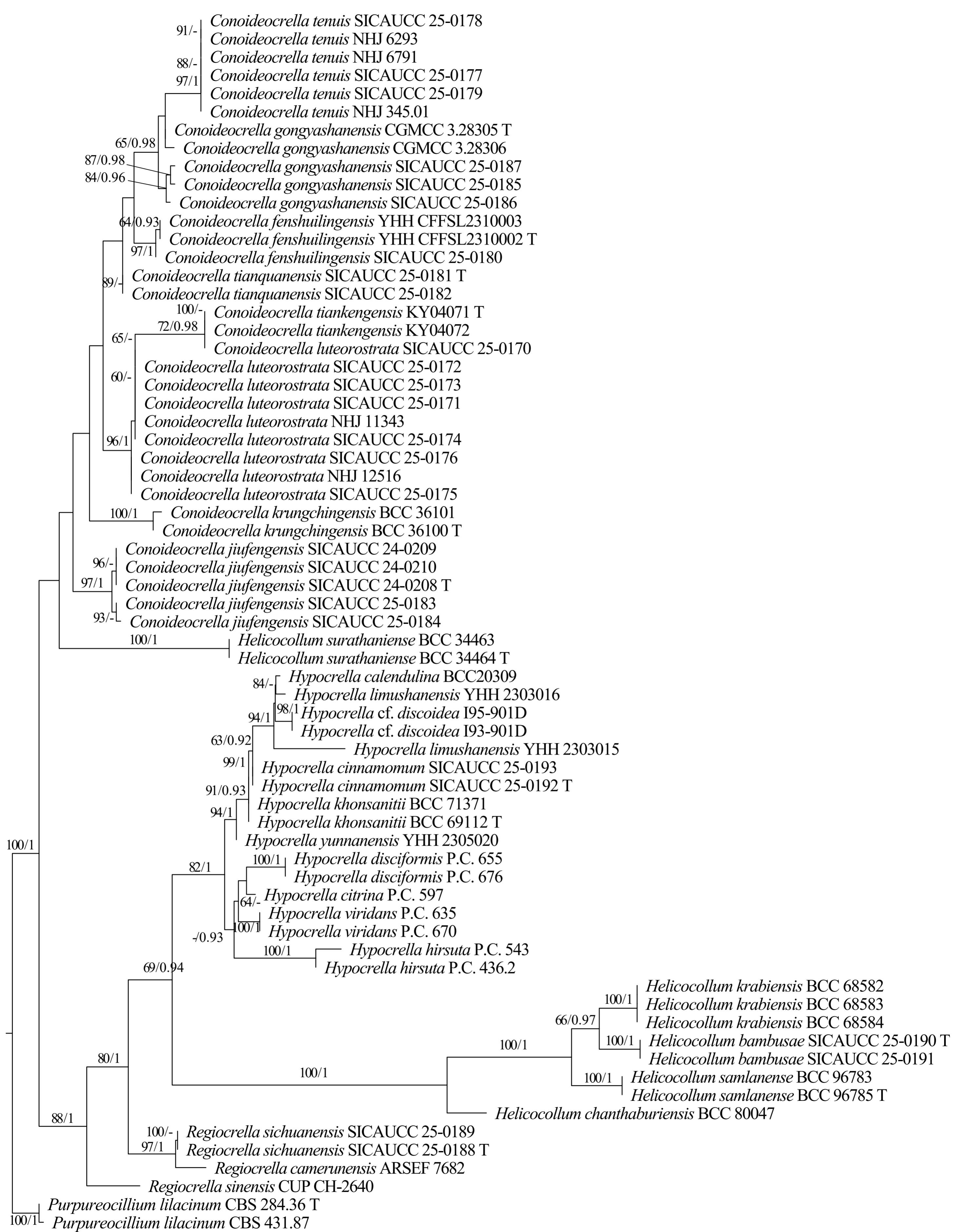

0.02

Supplement: Supplementary material 5 — Phylogenetic tree on LSU [file imafungus-17-e195753-s005.pdf]

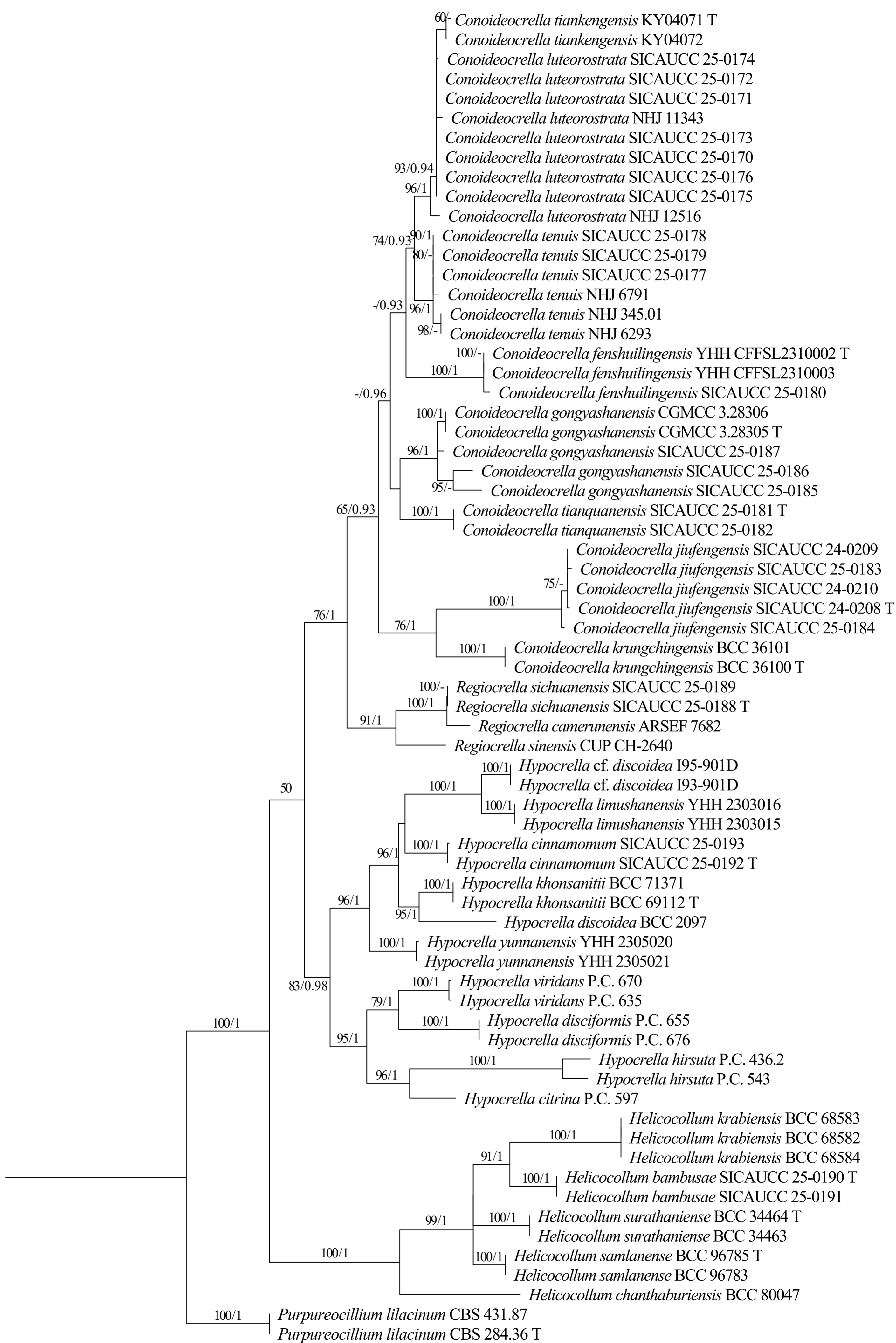

Supplement: Supplementary material 6 — Phylogenetic tree on tef1-α [file imafungus-17-e195753-s006.pdf]

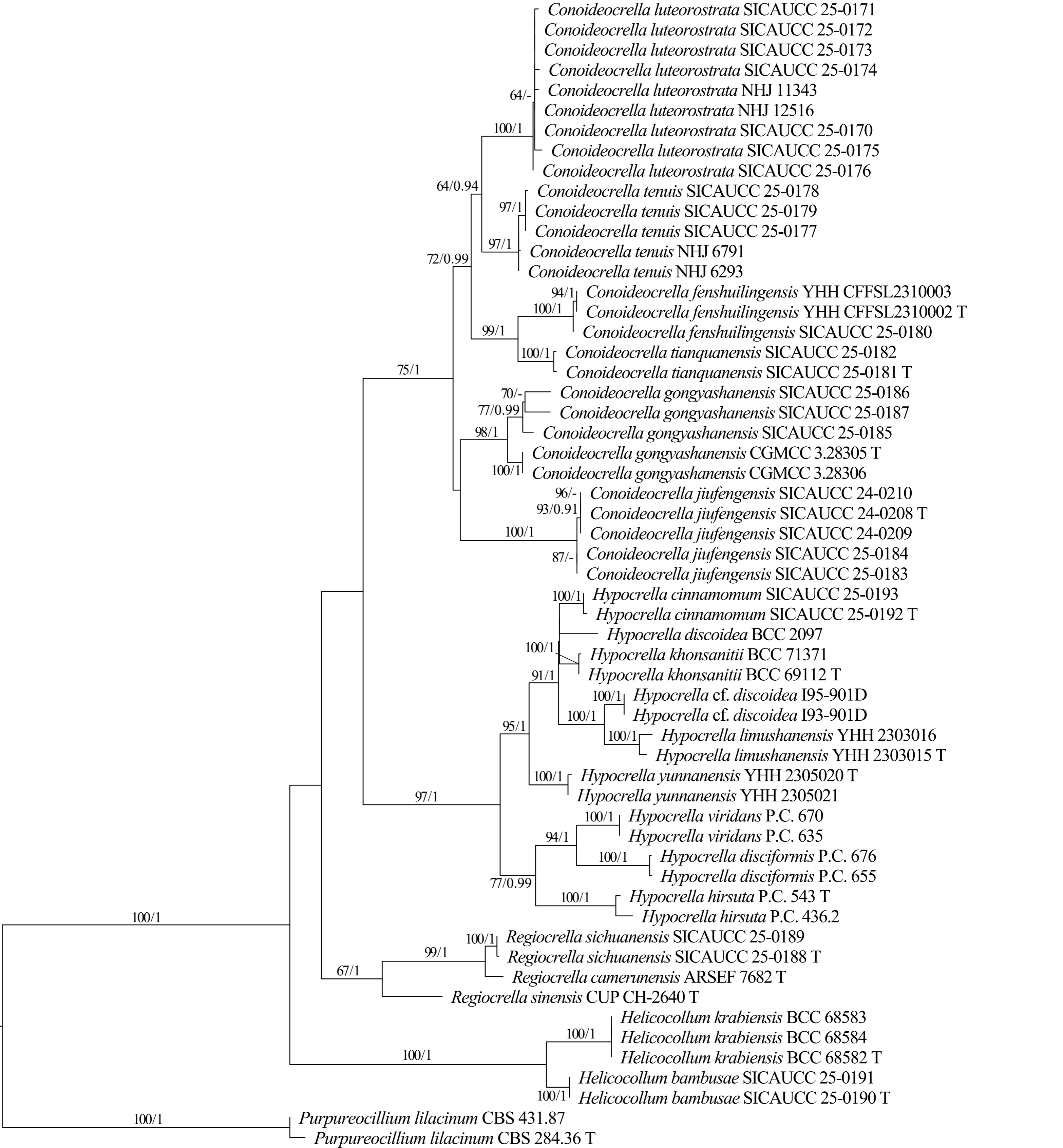

0.05

Supplement: Supplementary material 7 — Phylogenetic tree on rpb1 [file imafungus-17-e195753-s007.pdf]
